# Mitofusin2 Promotes *β* Cell Maturation from Mouse Embryonic Stem Cells via Sirt3/Idh2 Activation

**DOI:** 10.1155/2022/1172795

**Published:** 2022-03-27

**Authors:** Li Lu, Li Zhitao, Cui Nannan, Huang Mingzhu, Hu Xiaoping, Hong Dongsheng, Pan Zongfu, Lu Xiaoyang

**Affiliations:** ^1^The First Affiliated Hospital, Zhejiang University School of Medicine, Hangzhou, Zhejiang, China; ^2^Department of Pharmacy, Zhejiang Provincial People's Hospital, Hangzhou, Zhejiang, China

## Abstract

*β* cell dysfunction is the leading cause of diabetes. Adult *β* cells have matured glucose-stimulated insulin secretion (GSIS), whereas fetal and neonatal *β* cells are insensitive to glucose and are functionally immature. However, how *β* cells mature and acquire robust GSIS is not fully understood. Here, we explored the potential regulatory proteins of *β* cell maturation process and the capacity for GSIS. Combined with the data from public databases, we found that the gene expression of *Mitofusin2* (*Mfn2*) showed an increasing trend from mouse neonatal *β* cells to mature *β* cells. Moreover, its protein expression increased during mouse embryonic pancreas development and *β* cell differentiation from mouse embryonic stem cells. Knocking down *Mfn2* reduced Urocortin3 (Ucn3) expression, GSIS, and ATP production in induced *β* cells, while overexpressing it had the opposite effect. However, neither *Mfn2* knockdown nor overexpression affected the differentiation rate of insulin-positive cells. In immature and mature *β* cells, *Mfn2* and its correlated genes were enriched in tricarboxylic acid (TCA) cycle-related pathways. The expressions of Sirtuin 3 (*Sirt3*) and isocitrate dehydrogenase 2 (NADP+) and mitochondrial (*Idh2*) were *Mfn2*-regulated during *β* cell differentiation. Inhibiting Idh2 or Sirt3 reduced cellular ATP content and insulin secretion levels that increased by *Mfn2* overexpression. Thus, Mfn2 modulated the induced *β* cell GSIS by influencing the TCA cycle through Sirt3/Idh2 activation. We demonstrated that Mfn2 promoted embryonic stem cell-derived *β* cell maturation via the Sirt3/Idh2 pathway, providing new insights into *β* cell development. Our data contribute to understanding diabetes pathogenesis and offer potential new targets for *β* cell regeneration therapies.

## 1. Introduction

Diabetes is a common metabolic disorder characterized by hyperglycemia. The International Diabetes Federation reported that 463 million people were living with diabetes in 2019 (https://www.diabetesatlas.org). The pathogenesis of diabetes is complicated, and *β* cell dysfunction resulting in impaired insulin secretion function plays a vital role in both type 1 and type 2 diabetes [1]. Available medications treat but do not cure diabetes; thus, finding effective therapies is paramount. Restoring *β* cell insulin secretion in diabetic patients is one possible strategy.

Pancreatic *β* cell maturation occurs gradually during postnatal development [2, 3]. During *β* cell development, immature fetal and neonatal *β* cells are poorly responsive to glucose and have low glucose-stimulated insulin secretion (GSIS) [4–6]. As they mature, *β* cells acquire highly sensitive and robust GSIS. Dedifferentiation of mature *β* cells into immature *β* cells or progenitor cells contributes to the development of type 2 diabetes [7–10]. Moreover, differentiation of functional mature *β* cells from embryonic stem (ES) or induced pluripotent stem cells is possible approaches to replenishing the *β* cell pools [11]. However, *β* cells derived from stem cells possess limited GSIS, resembling fetal *β* cells [12, 13]. Therefore, exploring the underlying mechanisms of *β* cell maturation and their GSIS capacity would contribute to understanding the pathogenesis of diabetes and finding new targets for *β* cell regeneration therapy.

GSIS is driven by glucose stimulated ATP generation, which relies on the tricarboxylic acid cycle (TCA) cycle and oxidative phosphorylation [1]. Glycolysis predominates in fetal and neonatal *β* cells, while the mature *β* cells have an increased rate of oxidative glucose metabolism [14]. Hence, switching from anaerobic glycolysis to mitochondrial oxidative phosphorylation is key to enhancing *β* cell GSIS function [15]. Mitofusin2 (Mfn2) participates in mitochondrial fusion, contributing to the maintenance and operation of the mitochondrial network. We previously demonstrated that *Mfn2* knockdown in adult mouse islets causes impaired GSIS function [16]. *Mfn2* deficiency promoted the conversion of somatic cells to a pluripotent state and maintained it through restructuring mitochondrial dynamics and bioenergetics [17]. However, the role of Mfn2 in *β* cell differentiation and maturation from stem cells remains unclear.

Here, we demonstrated that Mfn2 promoted the maturation of mouse ES cell-derived *β* cells. It increased their ATP generation and GSIS through Sirtuin3 (Sirt3)/isocitrate dehydrogenase 2 (NADP+) and mitochondrial (Idh2) activation. Our results showed crucial aspects of the Mfn2/Sirt3/Idh2 pathway in the functional maturation of *β* cells and provide new insights into the mechanisms of *β* cell differentiation and regeneration.

## 2. Materials and Methods

### 2.1. Differentiation of *β* Cells from Mouse Embryonic Stem Cells

Mouse ES-D3 cells were kindly provided by the Stem Cell Bank, Chinese Academy of Sciences, and were induced to differentiate into *β* cells using a three-step protocol as previously described [18, 19]. Briefly, embryoid bodies (EBs) were formed by aggregating ES cells for 5 days. After 9 days in differentiation medium I, three germ layers were spontaneously generated from EBs. After 19 days in differentiation medium II, the cells were differentiated into the pancreatic lineage. The differentiation medium I composition was as follows: IMDM (Gibco, Waltham, MA, USA), 20% FBS (Gibco), Glutamax (Gibco), nonessential amino acids (Gibco), 450 *μ*M monothioglycerol (Sigma-Aldrich, St. Louis, MO, USA). Differentiation medium II consisted of DMEM/F12 (Gibco), 10 mM nicotinamide (Sigma-Aldrich), 1 *μ*g/mL laminin (Sigma-Aldrich), N2 media supplement (Gibco), and B27 media supplement (Gibco).

### 2.2. Knocking Down or Overexpressing *Mfn2* in Differentiated Cells

The lentivirus vector expressing short hairpin RNA (shRNA) targeting *Mfn2* (shRNA: GGAAGAGCACCGTGATCAA), the vector for overexpressing *Mfn2*, and their control were designed and synthesized by GeneChem (Shanghai, China). Differentiated cells on day 5 + 20 were cultured in a culture medium containing 5 *μ*g/mL polybrene (Sigma-Aldrich). They were infected with lentivirus at a multiplicity of infection (MOI) of 100 for 16 h and further cultured until day 5 + 28.

### 2.3. Chemical Treatment

Cells were treated with 10 *μ*mol/L Idh2 inhibitor AGI6780 (Selleck, Shanghai, China) or 50 *μ*mol/L Sirt3 inhibitor 3-TYP (Selleck) or mock-treated with DMSO from days 5 + 20 to 5 + 28.

### 2.4. Immunocytochemistry Analysis

Mouse ES cell-derived cells were fixed with methanol and blocked with 10% FBS. Cells were then incubated with the primary antibodies: anti-insulin (1 : 200; Cell Signaling Technology, Danvers, MA, USA) and anti-Mfn2 (1 : 100; Abcam, Cambridge, MA, USA) at 4°C overnight. The cells were treated with the appropriate secondary antibodies and DAPI (Sigma-Aldrich). The overlay images were merged using the Image-Pro Plus software.

### 2.5. Flow Cytometry Analysis

Mouse ES cell-derived cells on different differentiation days were digested into single cells with Accutase (Invitrogen, Waltham, MA, USA). The cells were fixed in 4% paraformaldehyde and then blocked with 3% BSA. After blocking, cells were incubated overnight with anti-insulin (1 : 500) at 4°C. The cells were treated with the appropriate secondary antibody and collected using a flow cytometer (Beckman Coulter, Carlsbad, CA, USA). The results were expressed as the percentage of fluorescence intensity.

### 2.6. Glucose Stimulated Insulin Secretion Analysis

The differentiated cells on different differentiation days were cultured without insulin for 3 h and were preincubated in Krebs-Ringer Bicarbonate HEPES buffer for 1 h. The medium was replaced with buffer containing 27.7 mM glucose or 5.5 mM glucose and incubated for 1 h. The supernatant was analyzed using the Rat/Mouse Insulin ELISA kit (Sigma-Aldrich). Released insulin was normalized to the total protein content. Due to the discrepancy in insulin-positive cell populations across different treatment groups, the insulin secretion level was defined as the ratio of the insulin secretion value to insulin-positive cell rates.

### 2.7. ATP and Lactate Production of Differentiated Cells

Differentiated cells were incubated with 2.5 mmol/L glucose for 1 h and analyzed using an ATP Bioluminescent Assay Kit (Sigma-Aldrich) or lactate assay kit (Sigma-Aldrich) according to the manufacturer's protocol.

### 2.8. Fetal Mouse Pancreas Collection

Balb/c mice were obtained from the Shanghai SLAC Laboratory Animal Co., Ltd. (Certificate SCXK2012-0002). Eight- to twelve-week-old Balb/c mice (4 females and 1 male) were housed under a 12 h light/dark cycle. The day on which vaginal sperm or copulation plug observed was defined as embryonic day 0 (E0). Mouse embryonic pancreases were collected from E13, E15, E17, E19, and newborns. The experiment is approved by the Animal Experimental Ethical Inspection of the First Affiliated Hospital, College of Medicine, Zhejiang University (No.2017-258).

### 2.9. Western Blot and Coimmunoprecipitation Analysis

Cell lysates were resolved by SDS-PAGE, transferred onto PVDF membranes, and incubated with the following primary antibodies: anti-Urocortin3 (Ucn3) (Santa Cruz Biotechnology, Dallas, Texas, USA), anti-Mfn2, anti-Idh2 (Abcam), anti-dehydrogenase E1 and transketolase domain containing 1 (Dhtkd1) (Abcam), anti-glutamate dehydrogenase 1 (Glud1) (Abcam), anti-Sirt3 (Abcam), and anti-GAPDH (Multisciences, Shanghai, China). The samples were incubated with horseradish peroxidase-conjugated secondary antibody. The blots were developed using enhanced chemiluminescence reagents (Affinity Biosciences, Cincinnati, OH, USA), and the density of the products was quantitated using the ImageJ software. For coimmunoprecipitation analysis, total protein extracts were immunoprecipitated with anti-Sirt3 antibody (ABclonal Technology, Hubei, China) and subjected to Western blot analysis by anti-Mfn2 antibody.

### 2.10. Bioinformatics Analysis of Mfn2 in Immature and Mature *β* Cells

The microarray dataset GSE54374 [20] and single-cell RNA-Seq dataset GSE87375 [21] containing *β* cells at postnatal day 0 (P0), P15, and adult (8-12 weeks or P60) were retrieved from the Gene Expression Omnibus (GEO, http://www.ncbi.nlm.nih.gov/geo/) database in the National Center for Biotechnology Information (NCBI). A series matrix file of GSE54374 and bulk cell RNA-Seq normalized gene transcripts per million (TPM) of GSE87375 were obtained for *Mfn2* expression profiles. The correlation between *Mfn2* and other genes in GSE54374 was calculated using the cor.test function by the R software at different time points. Genes with ∣*R* | >0.6 and *P* < 0.05 at each time point were set as genes correlated with *Mfn2*. Protein-protein interaction (PPI) networks of correlated genes at different time points were analyzed using the STRING database (http://string-db.org). The PPI network was then analyzed by DyNet [22], a Cytoscape plugin, to elucidate the dynamic molecular interaction networks of *Mfn2-*correlated genes during *β* cell maturation. DyNet dynamic rewiring score (Dn-score) performs an advanced analysis of the interactions associated with the nodes of multiple networks. A Dn − score > the first quartile of all correlated genes was identified as *Mfn2* highly correlated genes during *β* cell maturation. Gene ontology (GO) biological process and KEGG pathway annotations of highly correlated genes were analyzed using the ClusterProfiler package [23]. An adjusted *P* value < 0.05 was set as the cutoff criterion. Differentially expressed genes (DEGs) of GSE54374 between P0 *β* cells and 8-12-week *β* cells were analyzed using the GEO2R web tool (https://www.ncbi.nlm.nih.gov/geo/geo2r/). Genes were considered differentially expressed when the adjusted *P* value < 0.05 and ∣fold change | >1.5.

### 2.11. Real Time-Quantitative PCR Analysis

Total RNA was extracted from differentiated cells using RNAsimple Total RNA Kit (Tiangen, Beijing, China). cDNAs were extracted by PrimeScript RT reagent Kit (Takara Bio, Shiga, Japan), and the PCR amplifications were performed using TB Green Premix Ex Taq II (TaKaRa Bio). The relative expression levels were normalized to *Gapdh*. The following primer pairs were used: *Idh2*, forward 5′-TCCAGGAGATCTTTGACAAGCAC-3′, reverse 5′-ACAGATGTCATCAGGCCGAG-3′; *Dhtkd1*: forward 5′-ACGCCCCTCAGTTGACCAT-3′, reverse 5′-AAGCTGGGCGGTTTCGATAG-3′; *Glud1*: forward 5′-CTGCAACCATGTGTTGAGCC-3′, reverse 5′-ACCTCCAAACGGTACATCGAC-3′; *Gapdh*: 5′-GTCATCCATGACAACTTTGG-3′, reverse 5′-GAGCTTGACAAAGTGGTCGT-3′.

### 2.12. Activity of Idh2 in Differentiated Cells

Idh2 activity was quantified using the Idh assay kit (Sigma-Aldrich), following the manufacturer's instructions. Mitochondria were extracted using a mitochondrial extraction kit (Solarbio, Beijing, China), and the extracts were used to evaluate the Idh2 activity. One unit of Idh2 is the amount of enzyme that generates 1.0 *μ*mol of NADP per min at pH 8.0 and 37°C.

### 2.13. Molecular Docking

The protein 3D structures of Mfn2 and Sirt3 were obtained from the Protein Data Bank. They were optimized using UCSF Chimera (v.1.14) [24], and their structures were prepared for molecular docking with the Dock prep plugin. The docking was performed using the InterEvDock3 online server (http://bioserv.rpbs.univ-paris-diderot.fr/services/InterEvDock3/) [25], and the best model was selected based on the InterEvDock3 scoring function. ViewDock tool of UCSF Chimera was used to analyze the docking results.

### 2.14. Statistical Analysis

Data are presented as mean ± standard deviation (SD). Statistical analyses were performed using GraphPad Prism 8.3.0 (San Diego, CA, USA). Differences between two groups were assessed for significance using a two-tailed Student's *t*-test, whereas those between multiple groups were assessed by a one-way or two-way ANOVA.

## 3. Results

### 3.1. *β* Cell Differentiation and Maturation from Mouse Embryonic Stem Cell

**Figure 1:**
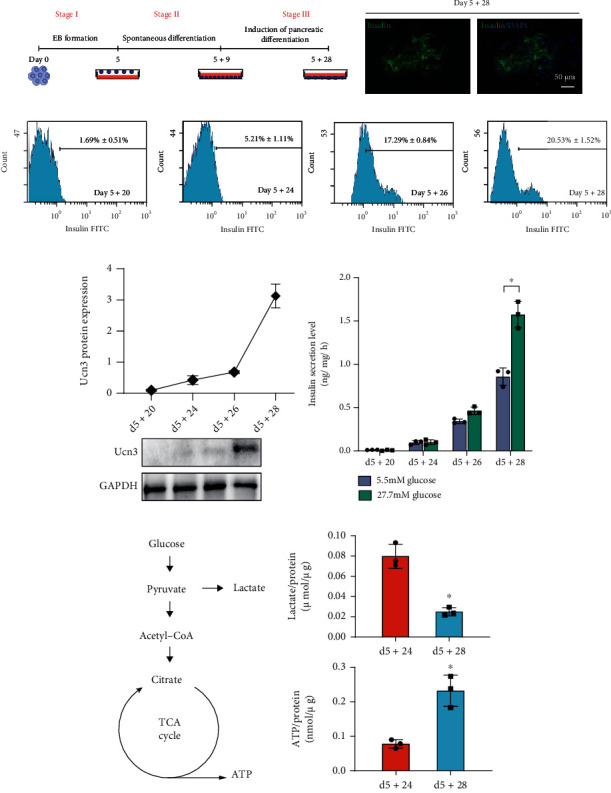
Differentiation of functional insulin cells from embryonic stem cells. (a) Three-stage protocol of insulin cell differentiation. Insulin expression determination in the induced cells at termination day by immunofluorescence stain. Bar = 50 *μ*m. (b) Flow cytometry assay demonstrated the ratios of insulin cells at different days. (c) Ucn3 protein expression during the insulin-positive cell generation period. (d) Released insulin of induced cells was analyzed by ELISA kit. (e) Lactate and ATP production after one-hour 2.5 mmol/L glucose stimulation. The lactate and ATP levels were compared to the total protein amount. Values were normalized with total protein contents. *n* = 3. Values represent mean ± S.D. ^∗^*P* < 0.05.

We differentiated *β* cells from mouse ES cells using a previously described three-step protocol (Figure 1(a)) [18, 19]. During the late period of the pancreatic differentiation stage, the ratio of insulin-positive *β* cells showed a robust, increasing trend (Figure 1(b)). The insulin-positive cells appeared on day 5 + 20, and their ratio was 1.69% ± 0.51%. As differentiation progressed, the ratio increased to 5.21% ± 1.11%, 17.29% ± 0.84%, and 20.53% ± 1.52% on days 5 + 24, 5 + 26, and 5 + 28, respectively (Figure 1(b)). We also detected the protein expression of the mature *β* cell marker Ucn3 during the period of insulin-positive cell generation to identify the differentiated insulin-positive cells. We found that Ucn3 was highly expressed on day 5 + 28 (Figure 1(c)), confirming the generation of mature *β* cells on the last day of differentiation.

Next, we assessed GSIS as a functional readout of the induced cells on different days (Figure 1(d)). The insulin levels of the induced cells on day 5 + 20 were not detected. The cells on day 5 + 24 seemed to be glucose responsive, but the insulin levels were low and not glucose concentration dependent. On day 5 + 26, the cells secreted insulin after glucose stimulation. Although the released insulin level increased numerically after high-concentration glucose incubation compared with that after low concentration, we observed no significant difference between the two groups. Nonetheless, the released insulin of differentiated cells on day 5 + 28 was significantly higher after stimulation with high concentration glucose compared with low concentration. These results indicated that the insulin-positive cells on day 5 + 24 or day 5 + 26 resemble immature *β* cells, which had deficient responses to glucose, while those on day 5 + 28 represent mature *β* cells.

Insulin secretion from *β* cells could be caused by glucose stimulated ATP production. Therefore, we analyzed the production of lactate and ATP at the immature (day 5 + 24) and mature *β* cell stage (day 5 + 28). Stimulating differentiated cells with 2.5 mmol/L glucose for 1 h revealed that the lactate level decreased and ATP production considerably rose on day 5 + 28 (Figure 1(e)). Thus, we speculated that glycolysis might be the dominant metabolic pathway in differentiated cells on day 5 + 24, while mitochondrial oxidative phosphorylation played a key role in differentiated cell on day 5 + 28.

### 3.2. Mfn2 Expression during *β* Cell Differentiation

**Figure 2:**
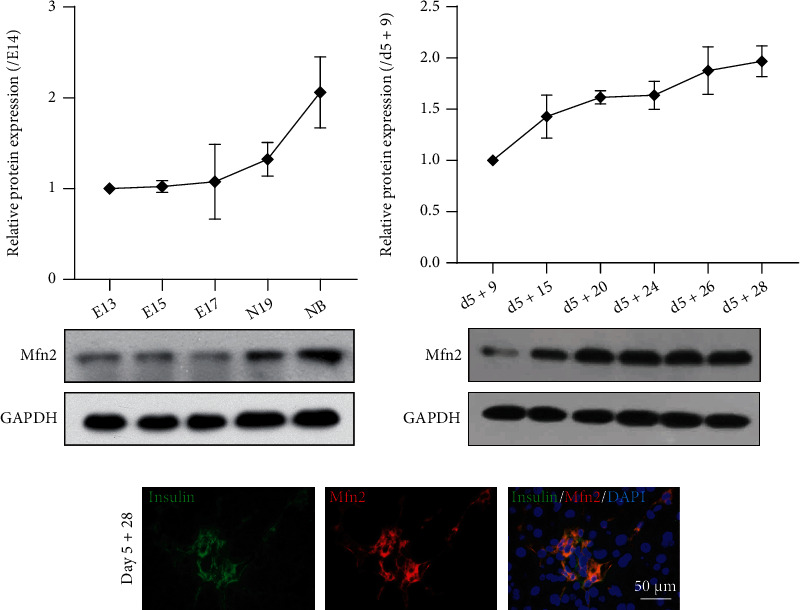
Mfn2 expression during pancreas maturation in vivo and in vitro. (a) Protein expression of Mfn2 in pancreas at embryonic days E13, 15, 17, and 19 of gestation and newborn mouse, *n* = 3. (b) Mfn2 expressed during *β* cell differentiation stage, *n* = 3. (c) Immunofluorescence staining detected the coexpression of Mfn2 with insulin at termination day 5 + 28. Bar = 50 *μ*m.

We first compared Mfn2 expression in the mouse embryonic pancreas in vivo to evaluate its expression during *β* cell development (Figure 2(a)). It remained almost at the same level from embryonic day E13 to E17 pancreas and displayed an increasing trend from E17 to the newborn pancreas stage. Next, we analyzed Mfn2 expression during the third *β* cell differentiation stage from days 5 + 9 to 5 + 28 (Figure 2(b)). We also demonstrated by immunofluorescence staining that almost all of the insulin-positive cells expressed Mfn2 on day 5 + 28 (Figure 2(c)).

### 3.3. Roles of Mfn2 in *β* Cell Differentiation and Maturation

**Figure 3:**
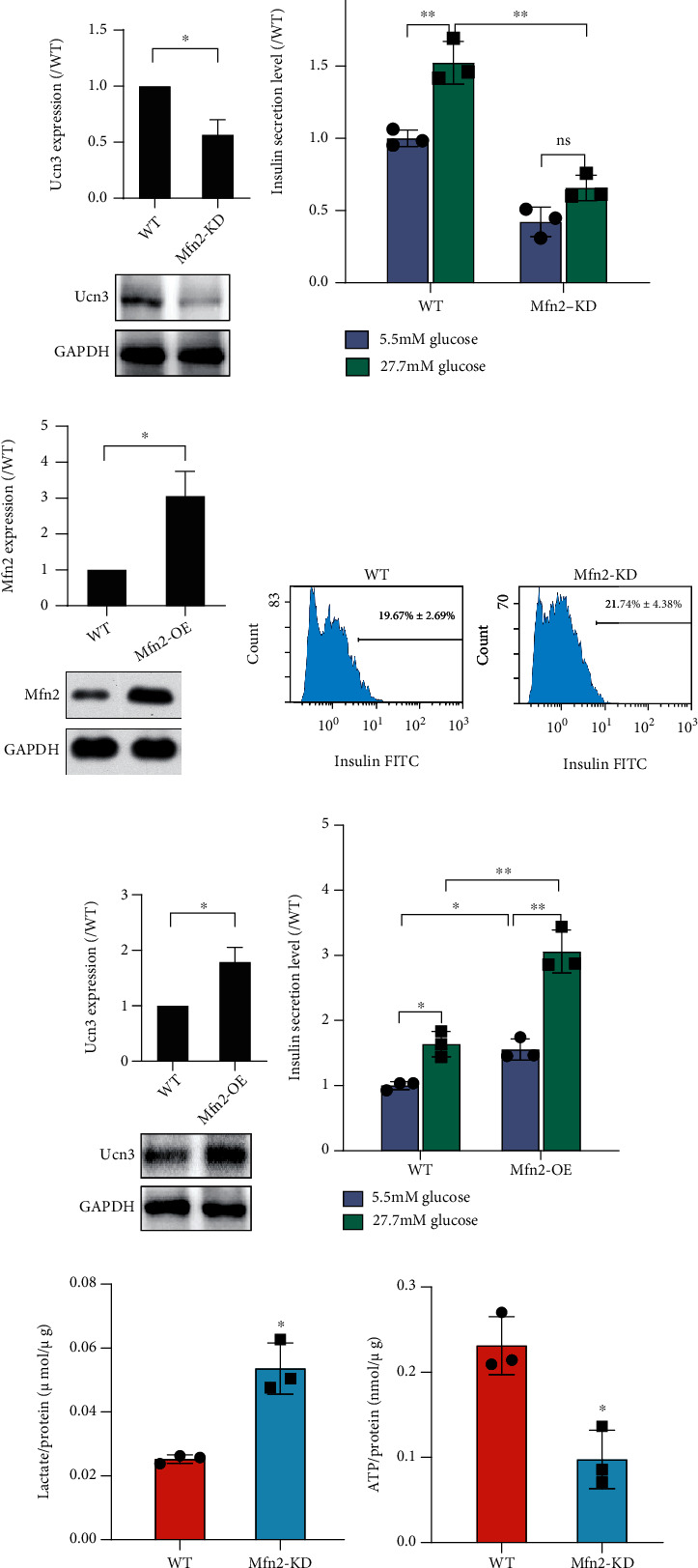
Effects of Mfn2 on *β* cell differentiation and GSIS function on day 5 + 28. (a) Mfn2 protein expression after *Mfn2* knockdown by shRNA infection. (b) Ratio of insulin-positive cells after *Mfn2* knockdown. (c) Ucn3 protein expression after *Mfn2* knockdown. (d) Insulin secretion level of *β* cells after *Mfn2* knockdown. (e) Mfn2 protein expression after *Mfn2* overexpression. (f) Ratio of insulin-positive cells after *Mfn2* overexpression. (g) Ucn3 protein expression after *Mfn2* overexpression. (h) Insulin secretion level of *β* cells after *Mfn2* overexpression. The insulin secretion level in each group was normalized to the insulin secretion level of WT cells stimulated with 5.5 mmol/L glucose. (i) Lactate production in *Mfn2* knockdown cells. (j) ATP production in *Mfn2* knockdown cells. (k) Lactate production in *Mfn2* overexpression cells. (l) ATP production in *Mfn2* overexpression cells. The lactate and ATP levels measured after one-hour 2.5 mmol/L glucose stimulation and were compared to the total protein amount. *n* = 3. The values represent mean ± S.D. Statistical significance was set as ^∗^*P* < 0.05, ^∗∗^*P* < 0.01. Student's *t*-test for (a–c, e–g, i–l); two-way ANOVA for (d, h).

Insulin-positive *β* cells appeared on day 5 + 20; therefore, we explored the effects of Mfn2 on the *β* cell generation and maturation. We used shRNA to knockdown *Mfn2* on day 5 + 20 and found that Mfn2 protein expression decreased to 35% upon silencing (Figure 3(a)). The ratio of insulin-positive cells after *Mfn2* knockdown (20.53% ± 2.87%) was similar to that of the control group (21.41% ± 1.43%) (Figure 3(b)). Ucn3 was also decreased after *Mfn2* knockdown (Figure 3(c)). Next, we evaluated GSIS of the induced insulin-positive cells. Because of the discrepancy in the ratios of insulin-positive cells, the insulin secretion level was calculated as the ratio of the released insulin to insulin-positive cell rates. Insulin secretion levels of the induced cells were significantly reduced after *Mfn2* knockdown than that of the control group. In addition, they were similar after low- or high-concentration glucose stimulation (Figure 3(d)). Thus, *Mfn2* knockdown rendered the differentiated cells unresponsive to glucose and conferred immature *β* cells.

We also used a lentivirus to overexpress *Mfn2* on day 5 + 20. Expression of Mfn2 increased to 2.8-fold compared with that of the control group on day 5 + 28 (Figure 3(e)). The ratio of induced insulin positive cells after *Mfn2* overexpression (21.74% ± 4.38%) did not significantly changed compared with the control group (19.67% ± 2.69%) (Figure 3(f)). Expression of Ucn3 was also enhanced following *Mfn2* overexpression (Figure 3(g)), indicating an increase of mature *β* cells. Moreover, ELISA showed that the insulin secretion levels of differentiated *β* cells increased after *Mfn2* overexpression (Figure 3(h)). These results implied that Mfn2 did not promote the differentiation but the maturation of induced insulin-positive cells.

Furthermore, we quantified lactate and ATP levels after *Mfn2* knockdown or overexpression. After glucose stimulation on day 5 + 28, *Mfn2* knockdown increased the cellular lactate level (Figure 3(i)) and decreased the ATP content (Figure 3(j)). The ATP content on day 5 + 24 was numerical increased without significance after *Mfn2* overexpression (supplementary Figure 1). Nonetheless, the overexpression reduced the lactate level (Figure 3(k)) and enhanced the ATP content (Figure 3(l)) in the differentiated cells on day 5 + 28. Hence, Mfn2 could alter the glucose metabolic pathways, modulating mature *β* cell insulin secretion.

### 3.4. *Mfn2*-Related Dynamic Networks in Mouse Immature and Mature *β* Cells

**Figure 4:**
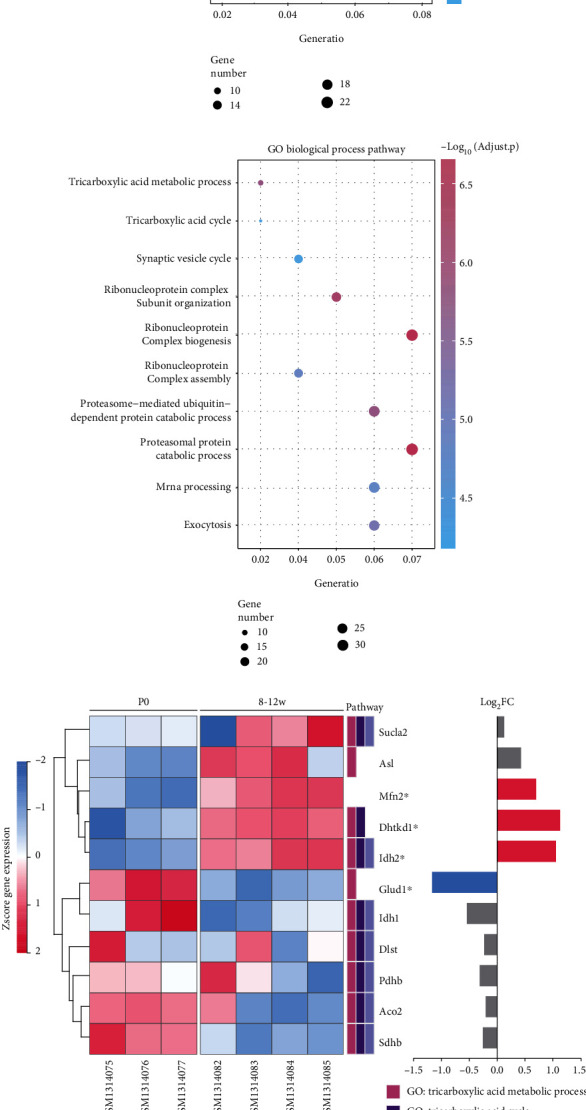
Bioinformatics analysis of Mfn2 in immature and mature *β* cells. (a, b) Mfn2 expression at different time points during *β* cell maturation in GSE54374 (a) and GSE87375 (b). (c, d) KEGG pathway (c) and GO biological process pathway (d) enrichment in highly correlated genes with Mfn2. The top 10 pathways ranked by adjust *P* values were listed in bubble charts. (e) Expression heat map of selected highly correlated genes and Mfn2. Genes with adjust *P* value < 0.05 and ∣fold change | >1.5 were set as significant DEGs. Blue charts indicated downregulated DEGs, and red charts indicated upregulated DEGs in 8-12-week *β* cells. (f) mRNA expressions of significant DEGs detected by qRT-PCR. (g) Protein expression detected by western blot. (h) Idh2 activity detected by Idh activity assay kit. *n* = 3. Data are means ± S.D. ^∗^*P* < 0.05 vs. day 5 + 24.

Since Mfn2 promoted *β* cell maturation during mouse embryonic stem cell differentiation, we analyzed its expression during the maturation process using the NCBI GEO public database. The transition from immature to mature *β* cells occurred between P9 and P15 [21]. In a microarray dataset GSE54374, expression of *Mfn2* was significantly higher in mature *β* cells (P15 and 8-12 weeks) than in the immature *β* cells (P0) (Figure 4(a)). It was also higher in mature *β* cells (P15 and P60) versus the immature (P0) at the bulk cell level in the single-cell RNA-seq dataset GSE87375 (Figure 4(b)). Correlated genes with *Mfn2* in GSE54374 dataset were then analyzed at different time points. Altogether, 1357 correlated genes at P0, 470 correlated genes at P15, and 1257 correlated genes at 8-12 weeks were identified. The PPI networks of correlated genes at P0, P15, and 8-12 weeks were constructed and further analyzed with DyNet plugin of Cytoscape. The DyNet central reference network contained 2102 nodes and 7155 edges. Consequently, 476 correlated genes with a Dn − score > 3 (the first quartile of all correlated genes) were identified as *Mfn2* highly correlated genes during *β* cell maturation (supplementary Table 1). Next, functional pathway enrichment of highly correlated genes with *Mfn2* was then analyzed. The top 10 GO biological process and KEGG pathways, as well as the enriched correlated genes, were listed (supplementary Table 2). We found that the KEGG pathway Citrate cycle (TCA cycle) (Figure 4(c)) and GO biological process pathways tricarboxylic acid metabolic process and tricarboxylic acid cycle (Figure 4(d)) were enriched in highly correlated genes. Altogether, 10 highly correlated genes were enriched in these three pathways. Their and *Mfn2* expression levels in the GSE54374 were presented as a heat map (Figure 4(e)). *Mfn2*, *Dhtkd1*, and *Ihd2* were upregulated DEGs between immature P0 and mature 8-12-week *β* cells, while *Glud1* was the downregulated DEG.

We then quantified the mRNA and protein expression of the three *Mfn2* correlated DEGs. We demonstrated that the mRNA level of *Idh2* was higher on day 5 + 28 than on day 5 + 24, while *Dhtkd1* and *Glud1* were constant during *β* cell differentiation (Figure 4(f)). Similarly, the protein expression of Idh2 also increased on the last day of *β* cell differentiation, while Dhtkd1 or Glud1 was unchanged (Figure 4(g)). Furthermore, we assessed the activity of Idh2 on different days and found that it was higher on day 5 + 28 than on day 5 + 24 (Figure 4(h)). The results showed that Idh2 expression and activation increase during the maturation of differentiated *β* cells.

### 3.5. Effects of *Mfn2* Knockdown or Overexpression on Idh2 in Differentiated *β* Cells

**Figure 5:**
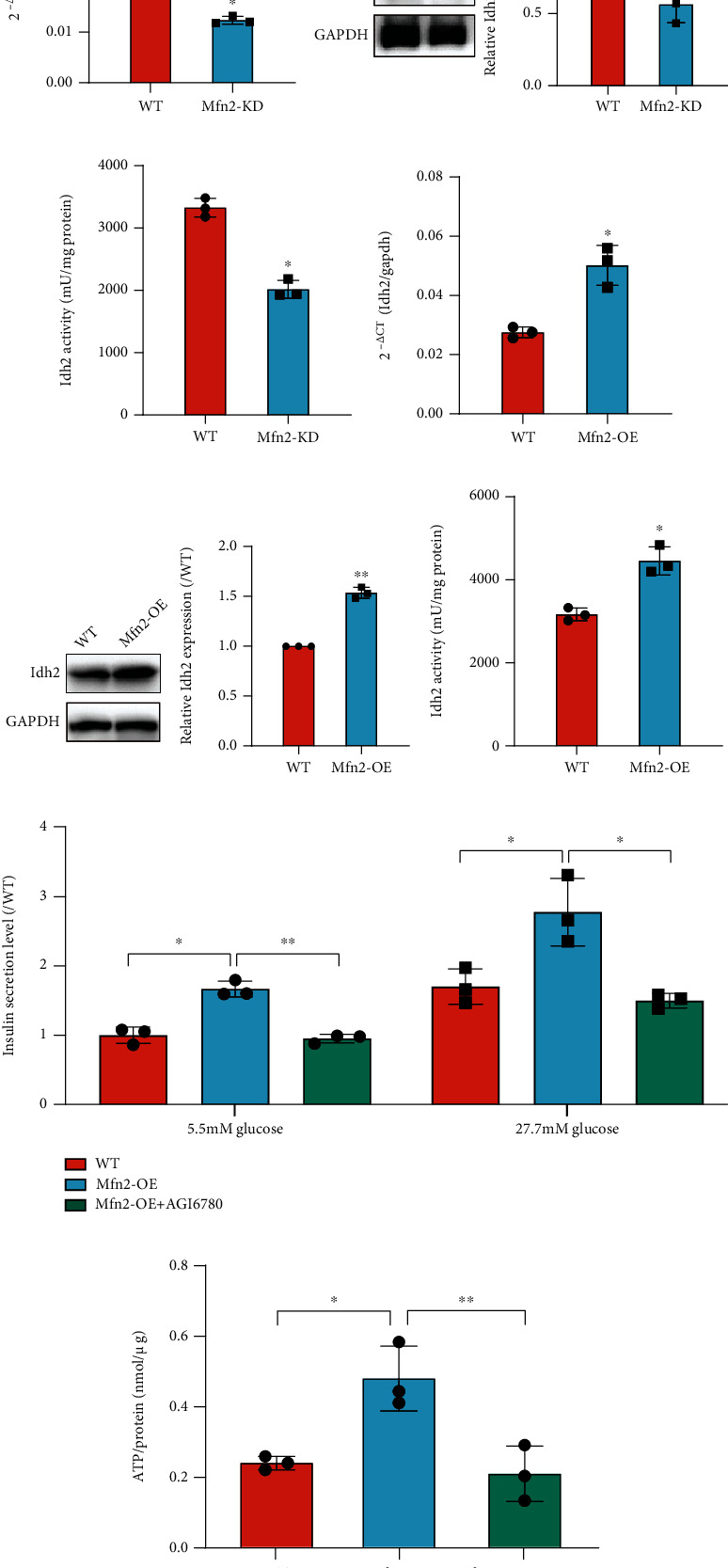
The effects of *Mfn2* knockdown and overexpression on Idh2 expression and activation. (a, d) *Idh2* mRNA expressions after *Mfn2* knockdown (a) or *Mfn2* overexpression (d) detected by qRT-PCR. (b, e) Idh2 protein expressions after *Mfn2* knockdown (b) or *Mfn2* overexpression (e) detected by western blot. (c, f) Idh2 activity after *Mfn2* knockdown (c) or *Mfn2* overexpression (f) detected by Idh activity assay kit. (g) Insulin secretion level of *β* cells was measured at termination day of differentiation after *Mfn2* overexpression with or without AGI6780 treatment. The insulin secretion level in each group was normalized to the insulin secretion level of WT cells stimulated with 5.5 mmol/L glucose. (h) ATP production on day 5 + 28 after one-hour 2.5 mmol/L glucose stimulation. The ATP levels were compared to the total protein amount. *n* = 3. Values represent mean ± S.D. Statistical significance was set as ^∗^*P* < 0.05 and ^∗∗^*P* < 0.01. Student's *t*-test for (a–f); one-way ANOVA for (h); two-way ANOVA for (g).

Idh2 plays a role in intermediary metabolism and energy production. We next analyzed the role of Mfn2 on Idh2 expression and activation. We analyzed how changes in Mfn2 expression affect Idh2 expression and protein activity. The *Idh2* mRNA level (Figure 5(a)) and protein expression level (Figure 5(b)) were both declined after *Mfn2* knockdown. Moreover, the activity of Idh2 decreased in *Mfn2* knockdown cells (Figure 5(c)). Conversely, upon Mfn2 overexpression, both the mRNA (Figure 5(d)) and protein levels (Figure 5(e)) increased. In agreement, the activity of Idh2 was also enhanced (Figure 5(f)).

To further explore the role of Idh2 in differentiated *β* cells, the Idh2 inhibitor AGI6780 which inhibits both mutated and wild-type protein [26] was applied to *Mfn2* overexpression cells from day 5 + 20. On day 5 + 28, GSIS was evaluated in the differentiated cells. AGI6780 significantly reduced *Mfn2* overexpression-induced high insulin levels after low- or high-concentration glucose stimulation (Figure 5(g)). It also reversed the *Mfn2* overexpression-enhanced ATP production caused by glucose stimulation (Figure 5(h)). These results demonstrated that Mfn2 may promote the *β* cell maturation via Idh2 activation.

### 3.6. Mfn2 Regulated *β* Cell Maturation through Sirt3/Idh2 Pathway

**Figure 6:**
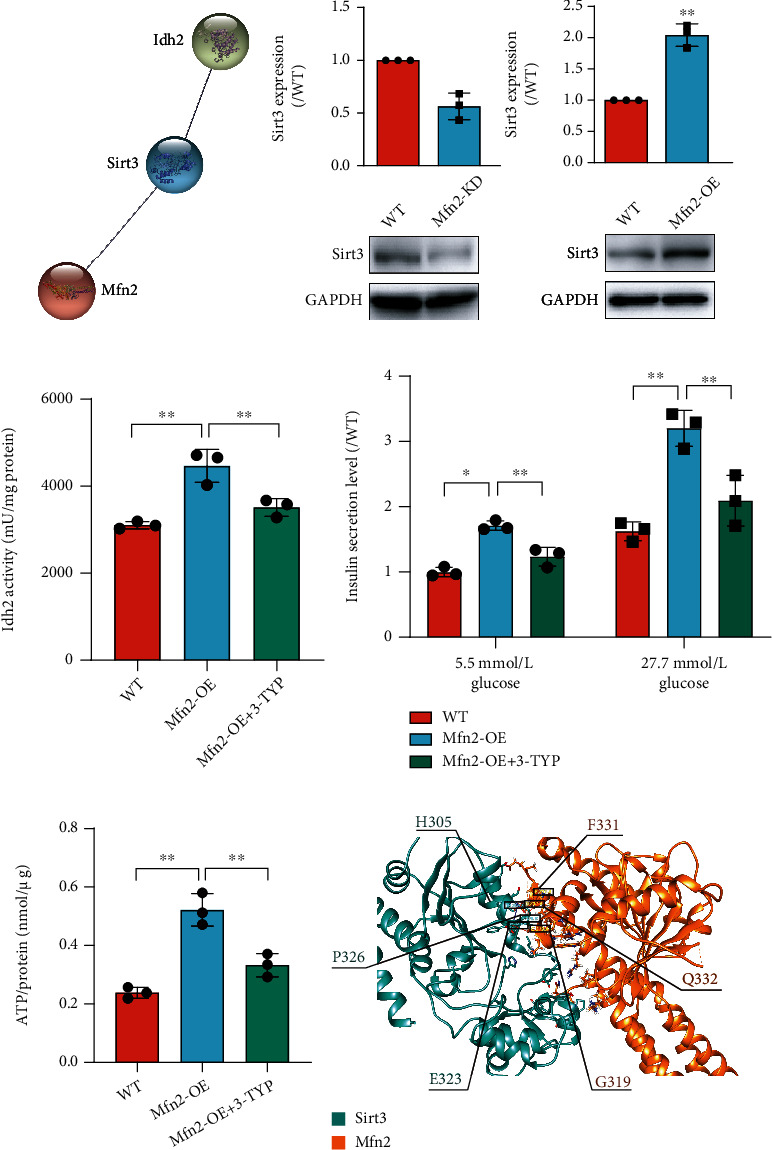
Sirt3 involved in Mfn2 promoted *β* cell maturation by regulating Idh2. (a) Potential interaction between Mfn2 and Idh2 was predicted by STRING database. (b) Protein expression levels of Sirt3 on day 5 + 28 after Mfn2 knockdown of overexpression. (c) Idh2 activity was detected by Idh activity assay kit at termination day of differentiation after Mfn2 overexpression with or without 3-TYP treatment. (d) Insulin secretion level of *β* cells was measured at termination day of differentiation after Mfn2 overexpression with or without 3-TYP treatment. The insulin secretion level in each group was normalized to the insulin secretion level of WT cells stimulated with 5.5 mmol/L glucose. (e) ATP production on day 5 + 28 after one-hour 2.5 mmol/L glucose stimulation. The ATP levels were compared to the total protein amount. *n* = 3. (f) Molecular docking analysis of Mfn2 and Sirt3. The green model represents Sirt3, and the yellow model represents Mfn2. Values represent mean ± S.D. Statistical significance was set as ^∗^*P* < 0.05 and ^∗∗^*P* < 0.01. Student's *t*-test for (b, c); one-way ANOVA for (e); two-way ANOVA for (d).

To explore how Mfn2 regulates Idh2 activation, we used the STRING database (https://string-db.org/) to identify potential interacting proteins. Indeed, the database predicted the protein-protein interactions among Mfn2, Sirt3, and Idh2 (Figure 6(a)). Therefore, we analyzed the effects of Sirt3 on *β* cell differentiation. Sirt3 is an NAD-dependent protein deacetylase that contributes to energy metabolism. We found that its expression reduced after *Mfn2* knockdown, whereas it increased after *Mfn2* overexpression (Figure 6(b)). To identify whether Mfn2 regulates Idh2 through Sirt3, we inhibited Sirt3 by a Sirt3 inhibitor 3-TYP in *Mfn2* overexpressing cells from day 5 + 20 during *β* cell differentiation. The increased Idh2 activity and ATP level caused by *Mfn2* overexpression was significantly suppressed by 3-TYP treatment (Figures 6(c) and 6(d)). Sirt3 inhibition by 3-TYP could also decrease the insulin secretion in *Mfn2* overexpressing cells after stimulation with low or high glucose (Figure 6(e)). Moreover, we analyzed the molecular docking of Mfn2 and Sirt3. We found that Mfn2 might directly interacted with Sirt3. The Mfn2 residues F331, Q332, and G319 and the Sirt3 residues H305, P326, and E323 were predicted to mediate the contact between the two proteins (Figure 6(f)). In addition, Sirt3 coimmunoprecipitated with Mfn2, indicating the potential interaction in differentiated cells (supplementary Figure 3). These data suggest that Sirt3/Idh2 regulates the insulin secretory ability in Mfn2-promoted *β* cell maturation.

## 4. Discussion

Loss of *β* cell GSIS is the main cause of diabetes. Considerable differences exist in the glucose metabolic pathways and GSIS between immature and mature *β* cells. Our study demonstrated that Mfn2 played an important role in the functional maturation of *β* cells differentiated from mouse ES cells. Exploring gene profiling datasets from public databases, we found that Mfn2 regulated GSIS and ATP production of the differentiated *β* cells through Sirt3 and Idh2 activation.

During *β* cell differentiation from mouse ES cells, insulin-positive cells first appeared on day 5 + 20. We found that the induced *β* cells on different differentiation days (days 5 + 24, 5 + 26, and 5 + 28) had inconsistent insulin responses to glucose. We inferred that this disparity was because of distinct glucose metabolic manners. Mitochondrial TCA cycle produces more ATP than glycolysis. In addition, glucose-derived ATP directly regulates the K_ATP_ channel activity, promoting insulin release [27]. Diabetes causes marked inhibition of mitochondrial metabolism in *β* cells, resulting in impaired GSIS [28]. Moreover, blunted mitochondrial function contributes to the immaturity of fetal and neonatal *β* cells [5]. Immature fetal and neonatal *β* cells favor anaerobic glycolysis, which occurs in the cytoplasm and results in increased lactate production, while mitochondrial TCA cycle and oxidative phosphorylation predominate in mature adult *β* cells [14, 29, 30]. Cells on day 5 + 24 had higher lactate levels and lower ATP levels after glucose stimulation, while differentiated cells on day 5 + 28 had lower glucose-induced lactate levels and higher ATP production. Additionally, Ucn3 is a confident marker for distinguishing between immature insulin-positive cells from functional mature *β* cells [31]. We found that Ucn3 expressed at a fairly low level on days 5 + 24 and 5 + 26 but high on day 5 + 28. These results indicated that differentiated *β* cells resembled immature fetal or neonatal *β* cells on day 5 + 24 and were more like mature *β* cells on day 5 + 28.

The present study elucidated the important role of Mfn2 in functional *β* cell differentiation. Mfn2 localizes at the mitochondrial membrane and in the cytosol and drives mitochondrial fusion [32]. We found that *Mfn2* expression showed an increasing trend from mouse neonatal *β* cells (P0) to mature *β* cells (P15, P60, or 8-12 weeks) in the public datasets GSE54374 [20] and GSE87375 [21]. We demonstrated that Mfn2 protein expression increased during mouse embryo pancreas development in vivo and during *β* cell differentiation from mouse ES cells in vitro. Mfn2 was also highly expressed in insulin-positive cells. Interestingly, *Mfn2* knockdown or overexpression from the day insulin-positive cells first appeared did not change the differentiation rate of insulin-positive cells but promoted the maturation of induced *β* cells. Overexpressing *Mfn2* enhanced Ucn3 expression and glucose-induced insulin release, accompanied by decreased lactate level and increased ATP content. By contrast, *Mfn2* knockdown elevated lactate level and declined the Ucn3 expression, ATP production, and insulin secretion level stimulated by different glucose concentrations. Furthermore, *Mfn2*-knockdown *β* cells were not glucose-responsive, and their patterns of insulin release were closer to that of immature *β* cells. This result agrees with our previous study, in which *Mfn2* was knocked down in isolated adult islets [16].

Generally, pluripotent cells rely heavily on anaerobic glycolysis, whereas differentiated mature cells rely on mitochondrial oxidative phosphorylation [33, 34]. Knockdown of *Mfn2* induced impaired mitochondrial fusion, decreasing mitochondrial oxidative phosphorylation [35]. Functional Mfn2 is crucial for embryonic development. Mouse ES cells maintain the pluripotency in the presence of leukemia inhibitory factor, and its withdrawal induced spontaneous differentiation of ES cells, accompanied by elevated Mfn2 expression [36]. Mfn2 mutant embryos exhibit specific and severe disruption of the mice placental trophoblast giant cell layer [37]. Low Mfn2 expression in vitro, by contrast, attenuated the blastocyst formation and preimplantation embryo development [38]. Moreover, Mfn2 deficiency dramatically enhances major enzymes involved in glycolysis and intracellular lactate production, reverting somatic cells into pluripotent cells [17]. Lactate augmentation suggested that glucose-derived pyruvate shifted away from the mitochondria [39]. In differentiated cells, *Mfn2* knockdown-induced lactate accumulation and ATP weakening imply mitochondrial dysfunction. The drop in anaerobic glycolysis enzymes, such as lactate dehydrogenase A, in mature *β* cells is believed to be critical for coupling glucose metabolism to insulin secretion [14]. We suggested that Mfn2 switched glucose anaerobic glycolysis to aerobic oxidation, promoting functional *β* cell maturation.

We demonstrated that *Mfn2* and its correlated genes in immature and mature *β* cells were enriched in TCA cycle-related pathways. Oxidative phosphorylation depends on the activity of dehydrogenase enzyme in the TCA cycle [32]. The *Mfn2-*correlated gene *Idh2* was involved in Mfn2-regulated GSIS in differentiated *β* cells. Reductive TCA cycle flux via Idh2 is required for GSIS, and Idh2 inhibition impairs GSIS in mice [26]. The role of Idh2 in pancreatic islet development has not been fully explored. We found that Mfn2 regulated Idh2 expression and activity. Inhibiting Idh2 reduced cellular ATP content and insulin secretion levels enhanced by *Mfn2* overexpression. We suggested that Mfn2 modulated GSIS of induced *β* cells by influencing the TCA cycle via Idh2.

Our bioinformatic analysis revealed interactions among Mfn2, Idh2, and Sirt3. Overexpressing *Mfn2* increased ATP production and activated Sirt3 in mouse neuroblastoma N2a cells [40]. Furthermore, Sirt3 could enhance Idh2 activity [41–43]. Sirt3 expression was markedly decreased in islets of patients with type 2 diabetes [44]. Knocking out *Sirt3* in *β* cells caused blunted insulin secretion [45], while overexpressing it reversed palmitate-induced pancreatic *β* cell apoptosis and enhanced GSIS [46, 47]. Until recently, little was known about the role of Sirt3 in pancreatic development. A recent study showed that prenatal testosterone exposure impaired insulin secretion in elderly female offspring via suppressing Sirt3 [48]. These finding implies an underlying association between Sirt3 and *β* cell function during the embryonic period. We demonstrated that Mfn2 regulated Sirt3 during *β* cell differentiation. Inhibiting Sirt3 abolished the elevated Idh2 activity, enhanced GSIS, and increased ATP content caused by *Mfn2* overexpression.

## 5. Conclusion

We demonstrated that Mfn2 plays a key role in promoting *β* cell maturation from mouse ES cells via Sirt3/Idh2 activation. These results shed light on the potential mechanisms that influence the *β* cell maturation process and the capacity for GSIS. Our findings will improve our understanding of diabetes pathogenesis and suggest potential targets for *β* cell regeneration therapies.

## Data Availability

The datasets GSE54374 and GSE87375 can be retrieved from Gene Expression Omnibus (GEO, http://www.ncbi.nlm.nih.gov/geo/) database in the National Center for Biotechnology Information (NCBI).

## Supplementary Materials

Supplementary Materials supplementary Figure 1: ATP production after one-hour 2.5 mmol/L glucose stimulation in Mfn2 overexpression cells at day 5 + 24. Supplementary Figure 2: the effects of Idh2 inhibition on Sirt3 protein expression after Mfn2 overexpression. Supplementary Figure 3: coimmunoprecipitation indicated the interaction of Mfn2 and Sirt3. Supplementary Table 1: list of the Mfn2 correlated genes. Supplementary Table 2: the top 10 pathways enriched in GO biological process and KEGG.

## References

[1] Seino S., Shibasaki T., Minami K. (2011). Dynamics of insulin secretion and the clinical implications for obesity and diabetes. *The Journal of Clinical Investigation*.

[2] Blum B., Hrvatin S., Schuetz C., Bonal C., Rezania A., Melton D. A. (2012). Functional beta-cell maturation is marked by an increased glucose threshold and by expression of urocortin 3. *Nature Biotechnology*.

[3] Arda H. E., Li L., Tsai J. (2016). Age-dependent pancreatic gene regulation reveals mechanisms governing human *β* cell function. *Cell Metabolism*.

[4] Hellerstrom C., Swenne I. (1991). Functional maturation and proliferation of fetal pancreatic beta-cells. *Diabetes*.

[5] Lien Y. C., Won K. J., Simmons R. A. (2020). Transcriptomic and quantitative proteomic profiling reveals signaling pathways critical for pancreatic islet maturation. *Endocrinology*.

[6] Lavine R. L., Chick W. L., Like A. A., Makdisi T. W. (1971). Glucose tolerance and insulin secretion in neonatal and adult mice. *Diabetes*.

[7] Talchai C., Xuan S., Lin H. V., Sussel L., Accili D. (2012). Pancreatic *β* cell dedifferentiation as a mechanism of diabetic *β* cell failure. *Cell*.

[8] Dor Y., Glaser B. (2013). Beta-cell dedifferentiation and type 2 diabetes. *The New England Journal of Medicine*.

[9] Efrat S. (2019). Beta-cell dedifferentiation in type 2 diabetes: concise review. *Stem Cells*.

[10] Cinti F., Bouchi R., Kim-Muller J. Y. (2016). Evidence of *β*-cell dedifferentiation in human type 2 diabetes. *The Journal of Clinical Endocrinology and Metabolism*.

[11] Aguayo-Mazzucato C., Bonner-Weir S. (2018). Pancreatic *β* cell regeneration as a possible therapy for diabetes. *Cell Metabolism*.

[12] Hrvatin S., O'Donnell C. W., Deng F. (2014). Differentiated human stem cells resemble fetal, not adult, *β* cells. *Proceedings of the National Academy of Sciences of the United States of America*.

[13] Rezania A., Bruin J. E., Arora P. (2014). Reversal of diabetes with insulin-producing cells derived in vitro from human pluripotent stem cells. *Nature Biotechnology*.

[14] Gu C., Stein G. H., Pan N. (2010). Pancreatic *β* cells require neuroD to achieve and maintain functional maturity. *Cell Metabolism*.

[15] Nair G. G., Liu J. S., Russ H. A. (2019). Recapitulating endocrine cell clustering in culture promotes maturation of human stem-cell-derived *β* cells. *Nature Cell Biology*.

[16] Li L., Pan Z. F., Huang X. (2016). Junctophilin 3 expresses in pancreatic beta cells and is required for glucose-stimulated insulin secretion. *Cell Death & Disease*.

[17] Son M. J., Kwon Y., Son M. Y. (2015). Mitofusins deficiency elicits mitochondrial metabolic reprogramming to pluripotency. *Cell Death and Differentiation*.

[18] Schroeder I. S., Rolletschek A., Blyszczuk P., Kania G., Wobus A. M. (2006). Differentiation of mouse embryonic stem cells to insulin-producing cells. *Nature Protocols*.

[19] Li L., Li T., Zhang Y. (2015). Peroxisome proliferator-activated receptor *β* /*δ* activation is essential for modulating p-Foxo1/Foxo1 status in functional insulin-positive cell differentiation. *Cell Death & Disease*.

[20] Benitez C. M., Qu K., Sugiyama T. (2014). An integrated cell purification and genomics strategy reveals multiple regulators of pancreas development. *PLoS Genetics*.

[21] Qiu W. L., Zhang Y. W., Feng Y., Li L. C., Yang L., Xu C. R. (2017). Deciphering pancreatic islet *β* cell and *α* cell maturation pathways and characteristic features at the single-cell level. *Cell Metabolism*.

[22] Goenawan I. H., Bryan K., Lynn D. J. (2016). Dynet: visualization and analysis of dynamic molecular interaction networks. *Bioinformatics*.

[23] Yu G., Wang L. G., Han Y., He Q. Y. (2012). Clusterprofiler: an R package for comparing biological themes among gene clusters. *OMICS*.

[24] Pettersen E. F., Goddard T. D., Huang C. C. (2004). UCSF Chimera?A visualization system for exploratory research and analysis. *Journal of Computational Chemistry*.

[25] Quignot C., Postic G., Bret H. (2021). InterEvDock3: a combined template-based and free docking server with increased performance through explicit modeling of complex homologs and integration of covariation-based contact maps. *Nucleic Acids Research*.

[26] Zhang G. F., Jensen M. V., Gray S. M. (2021). Reductive TCA cycle metabolism fuels glutamine- and glucose-stimulated insulin secretion. *Cell Metabolism*.

[27] Ashcroft F. M., Rorsman P. (2012). Diabetes mellitus and the *β* cell: the last ten years. *Cell*.

[28] Haythorne E., Rohm M., van de Bunt M. (2019). Diabetes causes marked inhibition of mitochondrial metabolism in pancreatic *β*-cells. *Nature Communications*.

[29] Boschero A. C., Bordin S., Sener A., Malaisse W. J. (1990). D-glucose and l-leucine metabolism in neonatal and adult cultured rat pancreatic islets. *Molecular and Cellular Endocrinology*.

[30] Stolovich-Rain M., Enk J., Vikesa J. (2015). Weaning triggers a maturation step of pancreatic *β* cells. *Developmental Cell*.

[31] Huang J. L., Lee S., Hoek P., van der Meulen T., Van R., Huising M. O. (2020). Genetic deletion of urocortin 3 does not prevent functional maturation of beta cells. *The Journal of Endocrinology*.

[32] Delmotte P., Sieck G. C. (2020). Endoplasmic reticulum stress and mitochondrial function in airway smooth muscle. *Frontiers in Cell and Development Biology*.

[33] Ito K., Suda T. (2014). Metabolic requirements for the maintenance of self-renewing stem cells. *Nature Reviews. Molecular Cell Biology*.

[34] Folmes C. D., Nelson T. J., Martinez-Fernandez A. (2011). Somatic oxidative bioenergetics transitions into pluripotency-dependent glycolysis to facilitate nuclear reprogramming. *Cell Metabolism*.

[35] Yao C. H., Wang R., Wang Y., Kung C. P., Weber J. D., Patti G. J. (2019). Mitochondrial fusion supports increased oxidative phosphorylation during cell proliferation. *ELIFE*.

[36] Lee J. E., Seo B. J., Han M. J. (2020). Changes in the expression of mitochondrial morphology-related genes during the differentiation of murine embryonic stem cells. *Stem Cells International*.

[37] Chen H., Detmer S. A., Ewald A. J., Griffin E. E., Fraser S. E., Chan D. C. (2003). Mitofusins Mfn1 and Mfn2 coordinately regulate mitochondrial fusion and are essential for embryonic development. *The Journal of Cell Biology*.

[38] Zhao N., Zhang Y., Liu Q., Xiang W. (2015). Mfn2 affects embryo development via mitochondrial dysfunction and apoptosis. *PLoS One*.

[39] Dumortier O., Fabris G., Pisani D. F. (2020). Microrna-375 regulates glucose metabolism-related signaling for insulin secretion. *The Journal of Endocrinology*.

[40] Liu M., Li X., Huang D. (2020). Mfn2 overexpression attenuates cardio-cerebrovascular ischemia-reperfusion injury through mitochondrial fusion and activation of the AMPK/Sirt3 signaling. *Frontiers in Cell and Development Biology*.

[41] Someya S., Yu W., Hallows W. C. (2010). Sirt3 mediates reduction of oxidative damage and prevention of age-related hearing loss under caloric restriction. *Cell*.

[42] Bergaggio E., Riganti C., Garaffo G. (2019). Idh2 inhibition enhances proteasome inhibitor responsiveness in hematological malignancies. *Blood*.

[43] Yu W., Dittenhafer-Reed K. E., Denu J. M. (2012). SIRT3 protein deacetylates isocitrate dehydrogenase 2 (IDH2) and regulates mitochondrial redox status. *The Journal of Biological Chemistry*.

[44] Caton P. W., Richardson S. J., Kieswich J. (2013). Sirtuin 3 regulates mouse pancreatic beta cell function and is suppressed in pancreatic islets isolated from human type 2 diabetic patients. *Diabetologia*.

[45] De Marchi U., Galindo A. N., Thevenet J. (2019). Mitochondrial lysine deacetylation promotes energy metabolism and calcium signaling in insulin-secreting cells. *The FASEB Journal*.

[46] Zhang H. H., Ma X. J., Wu L. N. (2016). Sirtuin-3 (SIRT3) protects pancreatic *β*-cells from endoplasmic reticulum (er) stress-induced apoptosis and dysfunction. *Molecular and Cellular Biochemistry*.

[47] Kim M., Lee J. S., Oh J. E. (2015). SIRT3 overexpression attenuates palmitate-induced pancreatic *β*-cell dysfunction. *PLoS One*.

[48] Zhou Y., Gong M., Lu Y., Chen J., Ju R. (2021). Prenatal androgen excess impairs beta-cell function by decreased sirtuin 3 expression. *The Journal of Endocrinology*.

