# Supplementary material for: Mitofusin2 Promotes β Cell Maturation from Mouse Embryonic Stem Cells via Sirt3/Idh2 Activation
Source: Stem Cells Int. 2022 Mar 27;2022:1172795. doi: 10.1155/2022/1172795 (PMC8977338; doi:10.1155/2022/1172795)
Supplement: Supplementary Materials — supplementary Figure 1: ATP production after one-hour 2.5 mmol/L glucose stimulation in Mfn2 overexpression cells at day 5 + 24. Supplementary Figure 2: the effects of Idh2 inhibition on Sirt3 protein expression after Mfn2 overexpression. Supplementary Figure 3: coimmunoprecipitation indicated the interaction of Mfn2 and Sirt3. Supplementary Table 1: list of the Mfn2 correlated genes. Supplementary Table 2: the top 10 pathways enriched in GO biological process and KEGG. [file 1172795.f1.docx]

supplementary Figure.1


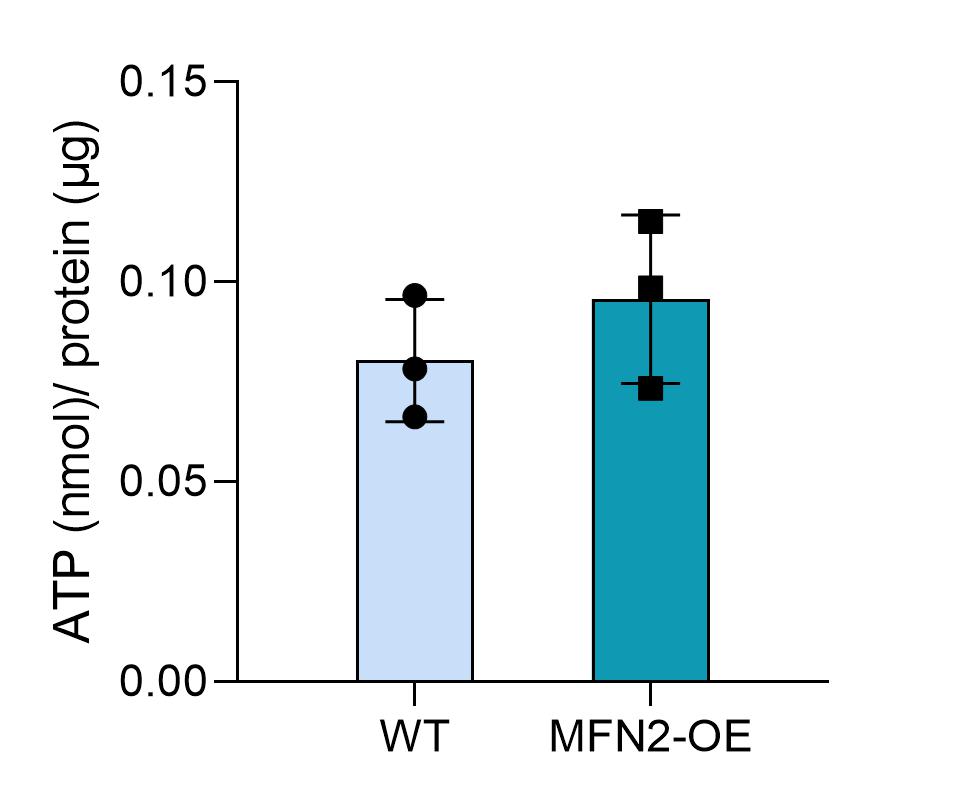


supplementary Figure.1. ATP production after one-hour 2.5mmol/L glucose stimulation in Mfn2 overexpression cells at day 5+24.

supplementary Figure.2


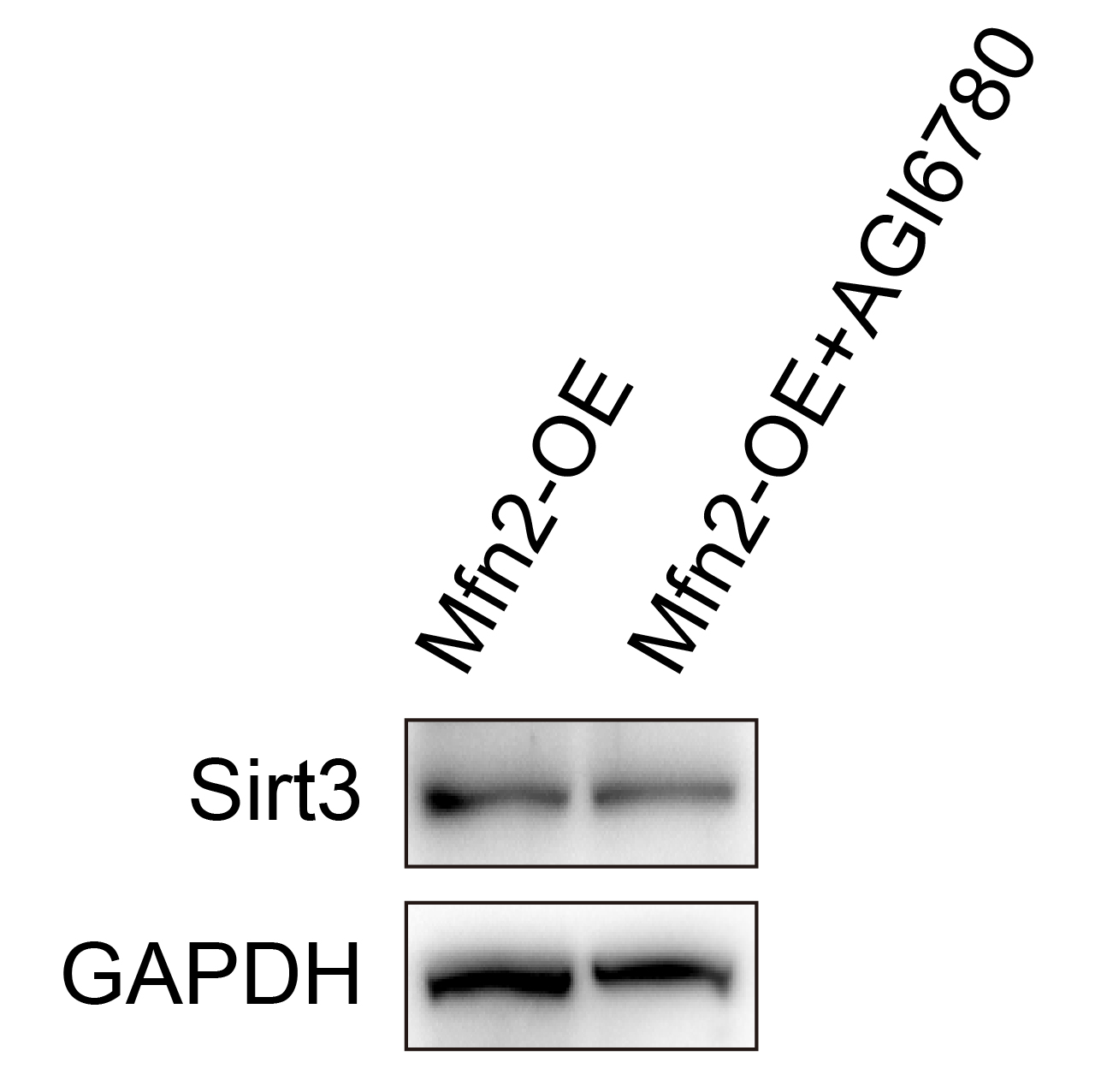


supplementary Figure.2. The effects of Idh2 inhibition on Sirt3 protein expression after Mfn2 overexpression.

supplementary Figure.3


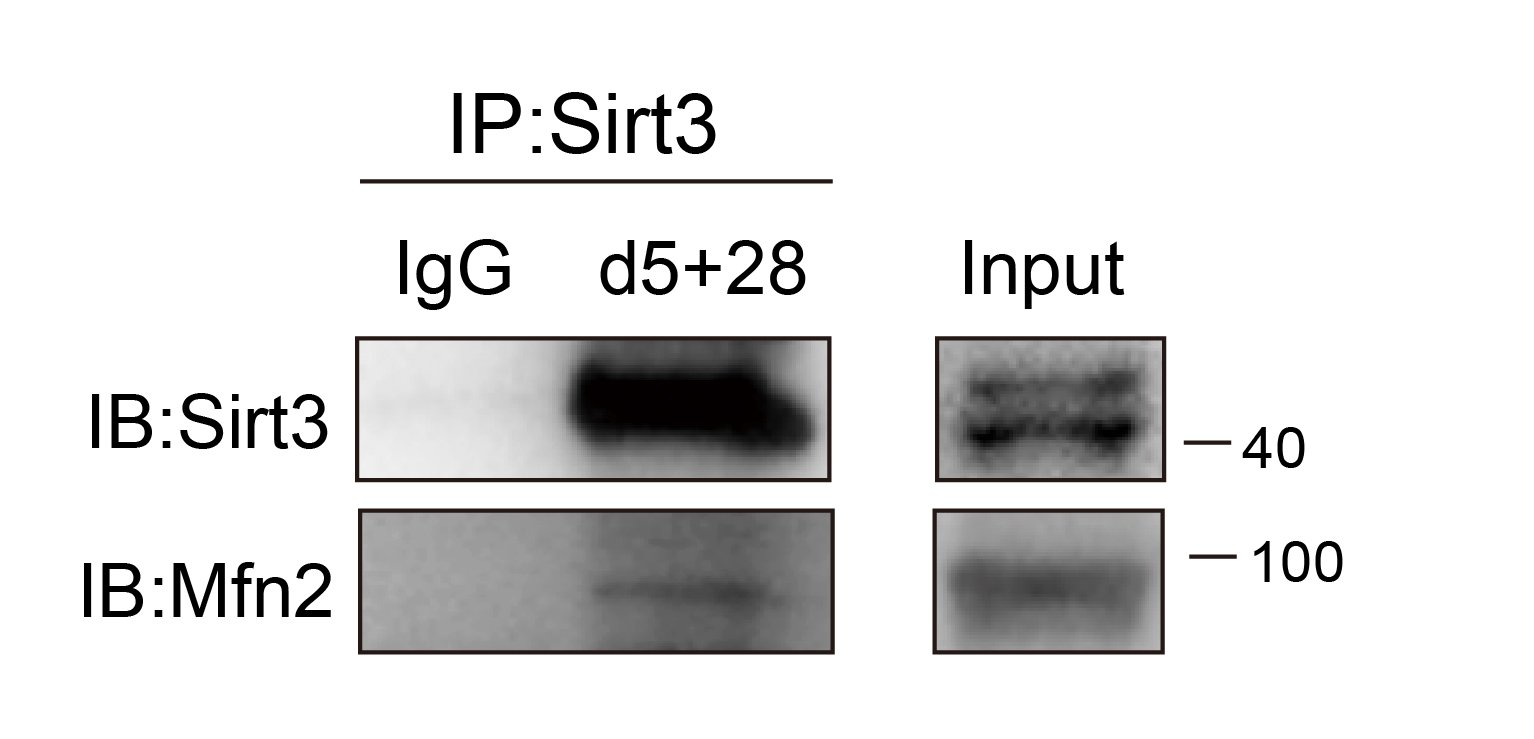


supplementary Figure.3. Co-immunoprecipitation indicated the interaction of Mfn2 and Sirt3.

supplementary Table.1 List of the *Mfn2* correlated genes

| name | Dn-score | logFC （8-12week vs P0） |
| --- | --- | --- |
| *Polr2e* | 25.66667 | -0.59040787 |
| *Psmd1* | 22.33333 | 0.10422751 |
| *Tnf* | 19.66667 | 1.07797415 |
| *Sdhb* | 15 | -0.25652416 |
| *Cdk2* | 15 | -2.35353546 |
| *Skp2* | 14.66667 | -3.19739144 |
| *Il18* | 14.33333 | 0.11966892 |
| *Hnrnpa2b1* | 14.33333 | -0.84509679 |
| *Ncbp2* | 14 | -0.78915958 |
| *Plrg1* | 13.66667 | -0.01031506 |
| *Dync1i2* | 13.66667 | -0.02918557 |
| *Prpf19* | 13 | -0.32471451 |
| *Mad2l1* | 13 | -3.17922782 |
| *Cdc27* | 13 | -0.27070985 |
| *Actb* | 13 | -0.61090649 |
| *Socs1* | 12.66667 | 0.20005623 |
| *Crebbp* | 12.33333 | -0.43497948 |
| *Prpf8* | 11.66667 | 0.18368987 |
| *Gnb1* | 11.66667 | -3.02608888 |
| *Copb1* | 11.66667 | -0.89717715 |
| *Brca1* | 11.33333 | -1.558702 |
| *Pa2g4* | 11 | -0.78182854 |
| *Anapc1* | 11 | -1.33770406 |
| *Rpl8* | 10.66667 | -0.34021858 |
| *Gusb* | 10.66667 | 0.32721392 |
| *Gng3* | 10.66667 | -2.52856511 |
| *Dlg4* | 10.66667 | 0.77554467 |
| *Tlr4* | 10.33333 | -1.02409517 |
| *Psmc4* | 10.33333 | -0.5852953 |
| *Huwe1* | 10.33333 | 1.17695093 |
| *Psmb4* | 10 | 0.09702694 |
| *Elavl1* | 10 | -1.3295057 |
| *Wwp1* | 9.666667 | -0.91955079 |
| *Uba1* | 9.666667 | 0.40436847 |
| *Tceb1* | 9.666667 | -0.26387888 |
| *Psmd14* | 9.666667 | -0.14053632 |
| *Atp5a1* | 9.666667 | -0.41013004 |
| *Aspm* | 9.666667 | -1.83708891 |
| *Nras* | 9.333333 | -1.61771398 |
| *Herc2* | 9.333333 | 0.46168903 |
| *Gpr17* | 9.333333 | -1.92754476 |
| *Utp3* | 9 | -0.29844806 |
| *Tnfsf11* | 9 | 0.3840767 |
| *Rpl18a* | 9 | -0.14721395 |
| *Rnf4* | 9 | -0.80949606 |
| *Ppp2r1b* | 9 | 0.15149107 |
| *Fbxo11* | 9 | -0.48957284 |
| *Dvl2* | 9 | -0.17913418 |
| *Csnk2a2* | 9 | -0.4728232 |
| *Ptpn11* | 8.666667 | 0.48701675 |
| *Psmd2* | 8.666667 | 0.21310656 |
| *Ppp2r5c* | 8.666667 | 1.05763755 |
| *Kctd7* | 8.666667 | 0.67065843 |
| *Cyc1* | 8.666667 | -0.42232242 |
| *Aurka* | 8.666667 | -1.8972209 |
| *Sucla2* | 8.666667 | -0.18197328 |
| *Prdx6b* | 8.666667 | 0.25896878 |
| *Gars* | 8.666667 | -0.14989055 |
| *Ube3b* | 8.333333 | -0.38912581 |
| *Ube2u* | 8.333333 | -0.59577112 |
| *Sf3b2* | 8.333333 | -0.63273002 |
| *Psmb6* | 8.333333 | -0.28387518 |
| *Myb* | 8.333333 | -1.42554831 |
| *Kbtbd8* | 8.333333 | 0.68425717 |
| *Hspa9* | 8.333333 | -0.40010398 |
| *Gata3* | 8.333333 | -0.85165825 |
| *Unkl* | 8 | -0.39290436 |
| *Ube2g2* | 8 | -0.33686043 |
| *Rps19* | 8 | -0.53019285 |
| *Rnf7* | 8 | -0.45668687 |
| *Nob1* | 8 | -1.19811927 |
| *Mfn2* | 8 | 0.70059722 |
| *Gria1* | 8 | -1.39883042 |
| *Fbxo41* | 8 | 0.9865676 |
| *Fbxo31* | 8 | 0.66327552 |
| *Prkcd* | 7.666667 | 0.71833728 |
| *Btbd1* | 7.666667 | -0.7486872 |
| *Zbtb16* | 7.666667 | -0.67480617 |
| *Mrps15* | 7.666667 | 0.94546909 |
| *Ywhab* | 7.333333 | -1.08585693 |
| *Rnf25* | 7.333333 | 0.53660322 |
| *Rnf182* | 7.333333 | 0.95692133 |
| *Men1* | 7.333333 | -0.71853106 |
| *Ktn1* | 7.333333 | -0.26169452 |
| *Kif5b* | 7.333333 | 0.86119413 |
| *Kif23* | 7.333333 | -2.08204528 |
| *Gdi2* | 7.333333 | -0.56169826 |
| *Fbxl8* | 7.333333 | 0.47537796 |
| *Ercc2* | 7.333333 | 0.34699817 |
| *Ddx27* | 7.333333 | -0.33775136 |
| *Upf3b* | 7 | 0.61876101 |
| *Uba2* | 7 | -1.32145985 |
| *Spdl1* | 7 | -1.62173697 |
| *Scp2* | 7 | -0.82527582 |
| *Psmb9* | 7 | 1.01358103 |
| *Psma6* | 7 | -0.4767393 |
| *Ppp2cb* | 7 | -0.99814136 |
| *Polr3a* | 7 | 0.31027026 |
| *Fbxw11* | 7 | 1.4982081 |
| *Cpsf1* | 7 | -0.44258062 |
| *Clasp1* | 7 | 0.93492472 |
| *Cd28* | 7 | -0.2843954 |
| *Cct7* | 7 | -0.2525572 |
| *Cct5* | 7 | -0.67585338 |
| *Ccna1* | 7 | -0.13893953 |
| *Bet1l* | 7 | 0.28197097 |
| *Plod2* | 7 | -3.25679617 |
| *Tubb4a* | 6.666667 | -0.23646319 |
| *Trrap* | 6.666667 | -0.99805677 |
| *Sptbn2* | 6.666667 | 1.99222349 |
| *Serpina3n* | 6.666667 | -0.65563362 |
| *Rps27* | 6.666667 | -0.52876578 |
| *Rps26* | 6.666667 | -0.96758248 |
| *Pcbp1* | 6.666667 | -0.70695416 |
| *Nup153* | 6.666667 | -1.30881361 |
| *Nudt21* | 6.666667 | -1.86641997 |
| *Gtpbp4* | 6.666667 | -0.4130305 |
| *Crp* | 6.666667 | 3.86526495 |
| *Bop1* | 6.666667 | 0.52002556 |
| *Becn1* | 6.666667 | 0.77248589 |
| *Atp5o* | 6.666667 | -0.47898974 |
| *Ago2* | 6.666667 | -0.86630661 |
| *Uba7* | 6.333333 | 0.38088272 |
| *Tsc2* | 6.333333 | 0.32986545 |
| *Trmt1* | 6.333333 | -0.38487489 |
| *Sqstm1* | 6.333333 | 0.68376885 |
| *Snrpb* | 6.333333 | -0.68242311 |
| *Smarca5* | 6.333333 | -0.44237999 |
| *Penk* | 6.333333 | -2.80748539 |
| *Ntrk2* | 6.333333 | 1.31512132 |
| *Myd88* | 6.333333 | -0.40103805 |
| *Kif20a* | 6.333333 | -2.5904344 |
| *Idh1* | 6.333333 | -0.54128119 |
| *Ddx39* | 6.333333 | -1.19315731 |
| *Cyfip1* | 6.333333 | -1.14465236 |
| *Ccar1* | 6.333333 | -0.28706042 |
| *Arid4b* | 6.333333 | -0.35658218 |
| *Taf3* | 6 | 1.01001962 |
| *Stx1a* | 6 | -1.15072566 |
| *Sf3b4* | 6 | -0.55673136 |
| *Psg23* | 6 | 0.78411613 |
| *Nup160* | 6 | -0.59138743 |
| *Ncor2* | 6 | 0.02704458 |
| *Lsm4* | 6 | -0.54365514 |
| *Kpnb1* | 6 | -2.49323577 |
| *Itga2* | 6 | -1.03526557 |
| *Isy1* | 6 | -0.59270421 |
| *Grm1* | 6 | 0.50479886 |
| *Gpc3* | 6 | -4.07665018 |
| *Hgh1* | 6 | 0.6592445 |
| *Dhx8* | 6 | -0.23492906 |
| *Ddx41* | 6 | 0.35310546 |
| *Ccp110* | 6 | -1.79921082 |
| *Capzb* | 6 | -0.94110364 |
| *Aurkaip1* | 6 | -0.16050577 |
| *Arpc4* | 6 | -1.40082846 |
| *Adcy9* | 6 | 1.62995779 |
| *Ube2j1* | 5.666667 | 0.65314304 |
| *Traf7* | 5.666667 | -1.40964407 |
| *Synj1* | 5.666667 | 1.67594193 |
| *Sst* | 5.666667 | 0.86916691 |
| *Snrpc* | 5.666667 | -0.81538305 |
| *Smarca4* | 5.666667 | -0.32422958 |
| *Scrib* | 5.666667 | -0.63456621 |
| *Psma8* | 5.666667 | -0.78808314 |
| *Prpf38a* | 5.666667 | -0.59539394 |
| *Prf1* | 5.666667 | 0.44801402 |
| *Ppil1* | 5.666667 | -1.48595676 |
| *Noc2l* | 5.666667 | 1.17296317 |
| *Ndufb5* | 5.666667 | -0.26809043 |
| *Mrpl39* | 5.666667 | -0.33298012 |
| *Lamb1* | 5.666667 | -4.71957474 |
| *Idh2* | 5.666667 | 1.05916939 |
| *Herc3* | 5.666667 | 0.26061819 |
| *Gan* | 5.666667 | 0.4621112 |
| *Dmd* | 5.666667 | -3.05054797 |
| *Cpeb1* | 5.666667 | 1.38966902 |
| *Col6a1* | 5.666667 | -5.68156293 |
| *Cav1* | 5.666667 | -5.18389488 |
| *Cars* | 5.666667 | 0.59668266 |
| *Avpr2* | 5.666667 | 0.43311555 |
| *Trim36* | 5.333333 | 0.46940632 |
| *Supt5* | 5.333333 | -0.08046729 |
| *Stag1* | 5.333333 | -1.23836375 |
| *Rpf1* | 5.333333 | -1.00386413 |
| *Qars* | 5.333333 | 1.06222382 |
| *Prkar2b* | 5.333333 | 0.64964675 |
| *Ppan* | 5.333333 | -0.56733291 |
| *Pla2g4e* | 5.333333 | 0.47636484 |
| *Pdhb* | 5.333333 | -0.3123314 |
| *Nphp1* | 5.333333 | 0.32740877 |
| *Kif3c* | 5.333333 | 0.45952589 |
| *Kdelr2* | 5.333333 | -0.71308377 |
| *Id2* | 5.333333 | -1.37908213 |
| *Htr1a* | 5.333333 | 0.41520309 |
| *Hecw2* | 5.333333 | -4.18336357 |
| *Grb2* | 5.333333 | 0.46958754 |
| *Gnai2* | 5.333333 | 0.07878311 |
| *Flcn* | 5.333333 | 0.70580086 |
| *Fbxl18* | 5.333333 | -0.04011915 |
| *Entpd1* | 5.333333 | -2.50511281 |
| *Eif3f* | 5.333333 | -0.66940083 |
| *Csf3* | 5.333333 | 0.34105566 |
| *Cbl* | 5.333333 | -0.82473364 |
| *Akt2* | 5.333333 | 0.56400744 |
| *Was* | 5 | 0.85856032 |
| *Vcam1* | 5 | -1.02483895 |
| *Usp25* | 5 | -0.82332284 |
| *Txndc5* | 5 | -0.62805617 |
| *Tubgcp2* | 5 | -0.65261842 |
| *Tfb2m* | 5 | 0.61734742 |
| *Rnf8* | 5 | 0.14986575 |
| *Rag1* | 5 | -0.000321 |
| *Nle1* | 5 | -0.15753315 |
| *Nfkbia* | 5 | 0.56404105 |
| *Mycn* | 5 | -3.05178219 |
| *Mrrf* | 5 | -0.02238139 |
| *Mrpl52* | 5 | -0.7608804 |
| *Mepe* | 5 | 0.24868953 |
| *Mchr1* | 5 | 0.10187564 |
| *Lox* | 5 | -7.41943227 |
| *Itpa* | 5 | -0.52285216 |
| *Ipo4* | 5 | -1.17796856 |
| *Igfbp5* | 5 | -2.41241504 |
| *Hmgcl* | 5 | -0.23880755 |
| *Hexb* | 5 | 0.34634262 |
| *Hectd3* | 5 | 1.25808026 |
| *Gsr* | 5 | -1.02525682 |
| *Gpx1* | 5 | 0.25784353 |
| *Glud1* | 5 | -1.16929442 |
| *Ghrhr* | 5 | 0.24371261 |
| *Gcgr* | 5 | 0.09312495 |
| *Fuca1* | 5 | 0.46634204 |
| *Fbxw22* | 5 | 0.29204817 |
| *Fancl* | 5 | -0.43636964 |
| *Dhx37* | 5 | 0.71474491 |
| *Dars* | 5 | -1.10506422 |
| *Cnih1* | 5 | -0.25859314 |
| *Cep57* | 5 | -0.6002722 |
| *Ccnt1* | 5 | 0.78429305 |
| *Brcc3* | 5 | 1.41898248 |
| *Atp5k* | 5 | -0.51621295 |
| *Asb17* | 5 | 0.22353472 |
| *Ap2m1* | 5 | 0.38190315 |
| *Xrcc3* | 4.666667 | 0.60779455 |
| *Ttf1* | 4.666667 | 1.03997453 |
| *Tbx5* | 4.666667 | 0.60819132 |
| *Scfd1* | 4.666667 | 1.14509135 |
| *Rpa3* | 4.666667 | -3.9279798 |
| *Rcn1* | 4.666667 | -1.68744861 |
| *Ptpn1* | 4.666667 | 0.77644819 |
| *Pias1* | 4.666667 | 0.76014843 |
| *P2ry13* | 4.666667 | 0.76415852 |
| *Ninl* | 4.666667 | -0.07207926 |
| *Ncapg* | 4.666667 | -2.69241708 |
| *4933400A11Rik* | 4.666667 | -0.11027034 |
| *Lyz1* | 4.666667 | 1.08808445 |
| *Kdm6b* | 4.666667 | -0.96651968 |
| *Ifnb1* | 4.666667 | 0.64652518 |
| *Ifih1* | 4.666667 | 1.06613583 |
| *Haus3* | 4.666667 | -0.76394862 |
| *Gtf2h4* | 4.666667 | -0.49701334 |
| *Dot1l* | 4.666667 | -0.64925443 |
| *Dnajc8* | 4.666667 | 0.10208091 |
| *Cyp2c29* | 4.666667 | 0.39637849 |
| *Cps1* | 4.666667 | -2.29245304 |
| *Cox4i1* | 4.666667 | -0.23081963 |
| *Cep72* | 4.666667 | -1.39842637 |
| *Atp5g1* | 4.666667 | -0.15090848 |
| *Zranb2* | 4.333333 | 0.37913458 |
| *Vti1b* | 4.333333 | 0.43144072 |
| *Txn1* | 4.333333 | -0.99166881 |
| *Tubgcp3* | 4.333333 | -0.99988926 |
| *Taf5* | 4.333333 | -0.55460998 |
| *Syn1* | 4.333333 | 1.73385477 |
| *Stx16* | 4.333333 | 0.20932736 |
| *Smg7* | 4.333333 | 0.50294101 |
| *Slc17a7* | 4.333333 | 0.17609098 |
| *Shisa5* | 4.333333 | 0.15581935 |
| *Rars2* | 4.333333 | -0.22622483 |
| *Puf60* | 4.333333 | -0.32823471 |
| *Psip1* | 4.333333 | 0.70423884 |
| *Prkaa2* | 4.333333 | 1.09409722 |
| *Pld2* | 4.333333 | -0.73929935 |
| *Pla2g4a* | 4.333333 | -2.09642138 |
| *Ndufc2* | 4.333333 | 0.57382398 |
| *Myh9* | 4.333333 | -0.76390985 |
| *Klc3* | 4.333333 | -0.65328981 |
| *Inpp5b* | 4.333333 | 0.48714673 |
| *Hnrnph2* | 4.333333 | -0.57089678 |
| *H2afy* | 4.333333 | 0.05943998 |
| *Gna14* | 4.333333 | 0.98842109 |
| *Gabrg2* | 4.333333 | 0.10002887 |
| *G6pc* | 4.333333 | 0.56463116 |
| *Entpd3* | 4.333333 | 0.29727414 |
| *Dvl3* | 4.333333 | 0.16352932 |
| *Ddx6* | 4.333333 | -1.05340472 |
| *Cxcr3* | 4.333333 | 0.50225335 |
| *Ctnnd1* | 4.333333 | 1.40779395 |
| *Col18a1* | 4.333333 | -2.18126887 |
| *Cep162* | 4.333333 | 1.30770361 |
| *Cask* | 4.333333 | 0.9979286 |
| *Cadm1* | 4.333333 | 0.78014015 |
| *Avpr1b* | 4.333333 | 0.19467161 |
| *Zc3h15* | 4 | 0.51566856 |
| *Xrn1* | 4 | 0.64567036 |
| *Wdr75* | 4 | -0.26090248 |
| *Wasf1* | 4 | -0.24239279 |
| *Tsg101* | 4 | -0.44259148 |
| *Tcf7* | 4 | -0.97380668 |
| *Stam* | 4 | -0.92419646 |
| *Socs6* | 4 | -0.73303105 |
| *Smarcc2* | 4 | -1.61536222 |
| *Smarcb1* | 4 | -0.87616798 |
| *Slu7* | 4 | 0.43989107 |
| *Serpine1* | 4 | -0.00449997 |
| *Serpinb8* | 4 | -0.24661685 |
| *Sec23a* | 4 | -0.55889243 |
| *Rras2* | 4 | -3.22380846 |
| *Rhob* | 4 | -1.62346246 |
| *Rbpj* | 4 | -1.11735643 |
| *Psg19* | 4 | 0.30862852 |
| *Pnrc2* | 4 | -0.6013607 |
| *Pgm5* | 4 | -3.29597354 |
| *Pex5l* | 4 | 0.80526363 |
| *Nup43* | 4 | -0.28603489 |
| *Myl3* | 4 | 0.39676937 |
| *Mrpl38* | 4 | 0.15244338 |
| *Mlst8* | 4 | 0.46719766 |
| *Mboat2* | 4 | -0.12894856 |
| *Kcnq2* | 4 | 0.58928897 |
| *H2-Ob* | 4 | 3.22826606 |
| *Gtf2e2* | 4 | -0.87956413 |
| *Gstk1* | 4 | -0.03053789 |
| *Gsdmd* | 4 | -1.11236135 |
| *Gns* | 4 | 1.42871864 |
| *Fshb* | 4 | 0.31104072 |
| *Frk* | 4 | 0.86695205 |
| *Fgg* | 4 | -1.58604635 |
| *Fam20c* | 4 | 0.68144049 |
| *Fabp1* | 4 | -1.54015949 |
| *Eif2s3x* | 4 | -1.95429971 |
| *Dmap1* | 4 | -0.57252087 |
| *Dlst* | 4 | -0.23120965 |
| *Col5a1* | 4 | -5.67320115 |
| *Col11a1* | 4 | -2.13532242 |
| *Cherp* | 4 | 1.6181161 |
| *Camk2a* | 4 | 0.50187588 |
| *Cacnb4* | 4 | 1.44655508 |
| *Brip1* | 4 | -1.86753396 |
| *Bard1* | 4 | -0.24443723 |
| *Azgp1* | 4 | 0.32885035 |
| *Atp5j2* | 4 | 0.50610155 |
| *Wnt7a* | 3.666667 | 0.52323603 |
| *Wnt4* | 3.666667 | 2.04039824 |
| *Tysnd1* | 3.666667 | 0.11575189 |
| *Tlr2* | 3.666667 | 1.08925393 |
| *Tkt* | 3.666667 | -0.29022469 |
| *Smarcal1* | 3.666667 | 1.22887831 |
| *Slco1b2* | 3.666667 | 0.52684599 |
| *Slc1a6* | 3.666667 | -0.1672628 |
| *Scg3* | 3.666667 | 0.29949143 |
| *Rock1* | 3.666667 | -0.42510261 |
| *Rhod* | 3.666667 | -1.47542487 |
| *Rheb* | 3.666667 | -0.44657858 |
| *Relb* | 3.666667 | 0.27401193 |
| *Rab3gap1* | 3.666667 | 1.029263 |
| *Pygm* | 3.666667 | -1.14987651 |
| *Ppy* | 3.666667 | 0.84364347 |
| *Polr3h* | 3.666667 | -0.13750381 |
| *Plcb2* | 3.666667 | 1.00775155 |
| *Plac8* | 3.666667 | -3.70429564 |
| *Oas1e* | 3.666667 | 0.33886052 |
| *Numb* | 3.666667 | -0.66951172 |
| *Nop10* | 3.666667 | -0.29207636 |
| *Nol12* | 3.666667 | -0.10383382 |
| *Ngfr* | 3.666667 | 0.91399172 |
| *Nbr1* | 3.666667 | 0.15012853 |
| *Msn* | 3.666667 | -3.29140127 |
| *Mrps24* | 3.666667 | 0.26516187 |
| *Lcn2* | 3.666667 | 0.68349281 |
| *Hspa12a* | 3.666667 | 1.09693101 |
| *Hira* | 3.666667 | 1.14996047 |
| *Gria3* | 3.666667 | -1.46859627 |
| *Glrx* | 3.666667 | -0.75415502 |
| *Gdap1* | 3.666667 | 2.20247943 |
| *Eif4a2* | 3.666667 | -0.99829001 |
| *Drg1* | 3.666667 | 0.58857082 |
| *Dpp4* | 3.666667 | 0.90104295 |
| *Dock2* | 3.666667 | 1.47037932 |
| *Dlgap1* | 3.666667 | 2.52887721 |
| *Cxcr6* | 3.666667 | -0.38753982 |
| *Cox6a2* | 3.666667 | 3.18325161 |
| *Cox15* | 3.666667 | -0.69733399 |
| *Col6a3* | 3.666667 | -3.89386767 |
| *Chrm1* | 3.666667 | 0.63932794 |
| *Cenpc1* | 3.666667 | -0.86435349 |
| *Casp3* | 3.666667 | -1.46460183 |
| *Btk* | 3.666667 | 0.81864284 |
| *Btbd3* | 3.666667 | -1.9158674 |
| *Bmpr1a* | 3.666667 | -0.69267929 |
| *B4galt7* | 3.666667 | 0.37340439 |
| *Aspscr1* | 3.666667 | 0.16743717 |
| *Arhgap35* | 3.666667 | 0.28954552 |
| *Amtn* | 3.666667 | 0.63798779 |
| *Adora1* | 3.666667 | 0.75130415 |
| *Aco2* | 3.666667 | -0.20941769 |
| *Zfp622* | 3.333333 | 1.40545695 |
| *Vdac3* | 3.333333 | -0.67646096 |
| *Uty* | 3.333333 | 3.35682079 |
| *Unc45a* | 3.333333 | 1.21737055 |
| *Ubqlnl* | 3.333333 | 0.54459895 |
| *Tpr* | 3.333333 | -0.8418973 |
| *Tmed3* | 3.333333 | 0.21029856 |
| *Tbc1d5* | 3.333333 | 1.7430433 |
| *Sv2a* | 3.333333 | 1.67744281 |
| *Stx4a* | 3.333333 | -0.19218602 |
| *Strn3* | 3.333333 | -1.10074661 |
| *Srsf10* | 3.333333 | -0.83366766 |
| *Sp7* | 3.333333 | -0.84030197 |
| *Sec22c* | 3.333333 | -0.84862015 |
| *Scarb2* | 3.333333 | 0.95132771 |
| *Sap30* | 3.333333 | -2.43166414 |
| *Rprd1a* | 3.333333 | 1.46761788 |
| *Rhof* | 3.333333 | 0.76886273 |
| *Ramp1* | 3.333333 | 0.58037793 |
| *Rab9* | 3.333333 | -1.38837602 |
| *Rab33b* | 3.333333 | 0.37406704 |
| *Rab18* | 3.333333 | -0.57512333 |
| *Ptgfr* | 3.333333 | 0.21259582 |
| *Prom1* | 3.333333 | 0.46136173 |
| *Prl* | 3.333333 | -0.16453131 |
| *Ppm1b* | 3.333333 | 0.53850676 |
| *Ppif* | 3.333333 | 0.69472362 |
| *Pmf1* | 3.333333 | -0.82364426 |
| *Pax7* | 3.333333 | 0.32447994 |
| *Nog* | 3.333333 | 0.04393415 |
| *Nlgn1* | 3.333333 | 0.63873528 |
| *Nfkbib* | 3.333333 | -0.07258477 |
| *Necap1* | 3.333333 | -0.08717433 |
| *Myh3* | 3.333333 | -0.14134116 |
| *Mybpc2* | 3.333333 | -0.50319756 |
| *Mx1* | 3.333333 | 0.56424957 |
| *Mrpl37* | 3.333333 | -0.29148828 |
| *Mis18bp1* | 3.333333 | -2.89742558 |
| *Mettl7a1* | 3.333333 | 1.12545368 |
| *Marcks* | 3.333333 | -1.62799215 |
| *Ipo8* | 3.333333 | 0.98977059 |
| *Il10ra* | 3.333333 | 2.46921327 |
| *Gpr15* | 3.333333 | -0.05192284 |
| *Gng8* | 3.333333 | -0.68095671 |
| *Gna12* | 3.333333 | -1.16050168 |
| *Gja1* | 3.333333 | -2.10782715 |
| *Ggt1* | 3.333333 | -0.47684616 |
| *Gap43* | 3.333333 | -1.2800358 |
| *Fermt3* | 3.333333 | 0.92474503 |
| *Exo1* | 3.333333 | -3.00157677 |
| *Epb41l5* | 3.333333 | 1.17030345 |
| *Dync2h1* | 3.333333 | -0.07425782 |
| *Drg2* | 3.333333 | -0.07360908 |
| *Dock1* | 3.333333 | -1.7065669 |
| *Dhtkd1* | 3.333333 | 1.13268685 |
| *Ctnna1* | 3.333333 | -0.83636251 |
| *Commd9* | 3.333333 | 0.50001671 |
| *Ckap2l* | 3.333333 | -1.76403897 |
| *Cep120* | 3.333333 | 0.18303483 |
| *Calcoco2* | 3.333333 | 0.24785765 |
| *Baz2a* | 3.333333 | 0.47999197 |
| *Atp5l* | 3.333333 | -0.55481326 |
| *Atg9a* | 3.333333 | -0.07440184 |
| *Atg4a* | 3.333333 | 0.22278144 |
| *Atg13* | 3.333333 | 1.28699814 |
| *Asl* | 3.333333 | 0.43148958 |
| *Adrb3* | 3.333333 | 0.23608029 |
| *Acp1* | 3.333333 | -1.33645701 |
| *Acan* | 3.333333 | -1.13483951 |

supplementary Table.2 The top 10 pathways enriched in GO biological process and KEGG.

| Description | p.adjust | gene |
| --- | --- | --- |
| GO biological process pathways | | |
| proteasomal protein catabolic process | 2.23E-07 | *Skp2/Prpf19/Anapc1/Psmb4/Wwp1/Psmd14/Herc2/Rnf4/Ppp2r5c/Psmd2/Aurka/Ube2u/Psmb6/Fbxo31/Ube2g2/Rnf7/Fbxl8/Psma6/Fbxw11/Ppp2cb/Psmb9/Cav1/Ube2j1/Psma8/Fbxl18/Hecw2/Usp25/Hectd3/Pias1/Socs6/Dlgap1/Gna12/Psmd1/Gpx1* |
| ribonucleoprotein complex biogenesis | 2.23E-07 | *Prpf19/Prpf8/Pa2g4/Rps19/Ddx27/Nudt21/Bop1/Gtpbp4/Ago2/Rps27/Snrpb/Lsm4/Isy1/Traf7/Noc2l/Snrpc/Rpf1/Ppan/Tfb2m/Ipo4/Nle1/Dhx37/Prkaa2/Psip1/Ddx6/Wdr75/Slu7/Eif2s3x/Nop10/Srsf10/Zfp622/Utp3/Nob1* |
| ribonucleoprotein complex subunit organization | 7.80E-07 | *Prpf19/Prpf8/Rps19/Kif5b/Nudt21/Bop1/Ago2/Rps27/Snrpb/Lsm4/Dhx8/Isy1/Traf7/Snrpc/Ppan/Mrrf/Nle1/Prkaa2/Psip1/Ddx6/Slu7/Eif2s3x/Srsf10* |
| tricarboxylic acid metabolic process | 2.04E-06 | *Idh1/Idh2/Pdhb/Glud1/Dlst/Aco2/Asl/Dhtkd1/Sdhb/Sucla2* |
| proteasome-mediated ubiquitin-dependent protein catabolic process | 2.04E-06 | *Skp2/Anapc1/Psmb4/Wwp1/Psmd14/Herc2/Rnf4/Ppp2r5c/Psmd2/Aurka/Ube2u/Psmb6/Fbxo31/Ube2g2/Rnf7/Fbxl8/Psma6/Fbxw11/Ppp2cb/Psmb9/Cav1/Ube2j1/Psma8/Fbxl18/Hecw2/Hectd3/Pias1/Gna12/Psmd1* |
| exocytosis | 9.79E-06 | *Clasp1/Sptbn2/Stx1a/Synj1/Htr1a/Gnai2/Gcgr/Scfd1/Pld2/Myh9/Cask/Stam/Camk2a/Cacnb4/Tsg101/Fgg/Rab3gap1/Glrx/Btk/Sv2a/Rab9/Nlgn1/Stx4a/Rab33b/Scrib/Pex5l/Wnt7a* |
| ribonucleoprotein complex assembly | 1.87E-05 | *Prpf19/Prpf8/Rps19/Nudt21/Bop1/Ago2/Rps27/Snrpb/Lsm4/Isy1/Traf7/Snrpc/Ppan/Nle1/Prkaa2/Psip1/Ddx6/Slu7/Eif2s3x/Srsf10* |
| mRNA processing | 2.22E-05 | *Ncbp2/Prpf19/Prpf8/Sf3b2/Cpsf1/Nudt21/Pcbp1/Ddx39/Snrpb/Aurkaip1/Lsm4/Sf3b4/Ddx41/Dhx8/Isy1/Ppil1/Prpf38a/Cpeb1/Snrpc/Supt5/Ccnt1/Psip1/Puf60/Slu7/Srsf10/Rprd1a/Hnrnpa2b1/Plrg1/Zranb2* |
| tricarboxylic acid cycle | 6.56E-05 | *Idh1/Idh2/Pdhb/Dlst/Aco2/Dhtkd1/Sdhb/Sucla2* |
| synaptic vesicle cycle | 6.56E-05 | *Actb/Sptbn2/Cyfip1/Stx1a/Synj1/Htr1a/Ap2m1/Syn1/Slc17a7/Cask/Camk2a/Cacnb4/Rab3gap1/Rock1/Sv2a/Nlgn1/Stx4a/Scrib/Wnt7a* |
| KEGG pathways | | |
| Ubiquitin mediated proteolysis | 1.97E-05 | *Skp2/Prpf19/Cdc27/Socs1/Brca1/Anapc1/Huwe1/Wwp1/Uba1/Herc2/Ube3b/Ube2u/Ube2g2/Rnf7/Rnf7/Fbxw11/Uba7/Herc3/Ube2j1/Cbl/Pias1/Fancl* |
| Spliceosome | 0.002118 | *Ncbp2/Prpf19/Prpf8/Sf3b2/Pcbp1/Snrpb/Lsm4/Sf3b4/Dhx8/Isy1/Ppil1/Prpf38a/Snrpc/Puf60/Cherp/Slu7/Srsf10/Plrg1* |
| Proteasome | 0.004231 | *Psmc4/Psmb4/Psmd14/Psmd2/Psmb6/Psma6/Psmb9/Psma8/Psmd1* |
| Toxoplasmosis | 0.004231 | *Tnf/Socs1/Tlr4/Myd88/Lamb1/Akt2/Gnai2/Nfkbia/Pla2g4a/Tlr2/Casp3/Ppif/Il10ra/Nfkbib/Pla2g4e/H2-Ob* |
| Long-term depression | 0.007167 | *Nras/Ppp2r1b/Gria1/Ppp2cb/Grm1/Gnai2/Pla2g4a/Plcb2/Gria3/Gna12/Pla2g4e* |
| Citrate cycle (TCA cycle) | 0.007444 | *Idh1/Idh2/Pdhb/Dlst/Aco2/Sdhb/Sucla2* |
| Chagas disease (American trypanosomiasis) | 0.008108 | *Tnf/Tlr4/Ppp2r1b/Ppp2cb/Myd88/Akt2/Gnai2/Nfkbia/Ifnb1/Gna14/Serpine1/Plcb2/Tlr2* |
| Oocyte meiosis | 0.022925 | *Mad2l1/Cdc27/Anapc1/Ppp2r1b/Ppp2r5c/Aurka/Ywhab/Fbxw11/Ppp2cb/Adcy9/Cpeb1/Camk2a/Cdk2* |
| Adherens junction | 0.023127 | *Actb/Crebbp/Was/Ptpn1/Ctnnd1/Wasf1/Ctnna1/Csnk2a2/Tcf7/Acp1* |
| Parkinson's disease | 0.030237 | *Uba1/Atp5a1/Cyc1/Ube2g2/Atp5o/Uba7/Ube2j1/Ndufb5/Atp5g1/Cox4i1/Ndufc2/Casp3/Cox6a2/Vdac3/Sdhb* |
